# Digital Phenotyping for Adolescent Mental Health: Feasibility Study Using Machine Learning to Predict Mental Health Risk From Active and Passive Smartphone Data

**DOI:** 10.2196/72501

**Published:** 2026-02-04

**Authors:** Balasundaram Kadirvelu, Teresa Bellido Bel, Aglaia Freccero, Martina Di Simplico, Dasha Nicholls, A Aldo Faisal

**Affiliations:** 1 Brain & Behaviour Lab, Department of Computing and Department of Bioengineering, Imperial College London, Royal School of Mines, London, SW72AZ, United Kingdom, 44 20 7594 6373; 2 Division of Psychiatry, Department of Brain Sciences, Imperial College London, London, England, United Kingdom; 3 Chair in Digital Health & Data Science, Faculty of Life Sciences, University of Bayreuth, Bayreuth, Bavaria, Germany

**Keywords:** youth mental health, digital health, mobile applications, mobile health, mHealth, smartphone sensing, ecological momentary assessment, EMA, artificial intelligence, early intervention

## Abstract

**Background:**

Adolescents are particularly vulnerable to mental disorders, with over 75% of lifetime cases emerging before the age of 25 years. Yet most young people with significant symptoms do not seek support. Digital phenotyping, leveraging active (self-reported) and passive (sensor-based) data from smartphones, offers a scalable, low-burden approach for early risk detection. Despite this potential, its application in school-going adolescents from general (nonclinical) populations remains limited, leaving a critical gap in community-based prevention efforts.

**Objective:**

This study evaluated the feasibility of using a smartphone app to predict mental health risks in nonclinical adolescents by integrating active and passive data streams within a machine learning (ML) framework. We examined the utility of this approach for identifying risks related to internalizing and externalizing difficulties, eating disorders, insomnia, and suicidal ideation.

**Methods:**

Participants (n=103; mean age 16.1 years, SD 1.0) from 3 UK secondary schools used the Mindcraft app (Brain and Behaviour Lab) for 14 days, providing daily self-reports (eg, mood, sleep, and loneliness) and continuous passive sensor data (eg, location, step count, and app usage). We developed a deep learning model incorporating contrastive pretraining with triplet margin loss to stabilize user-specific behavioral patterns, followed by supervised fine-tuning for binary classification of 4 mental health outcomes, namely, the Strengths and Difficulties Questionnaire (SDQ)-high risk, insomnia, suicidal ideation, and eating disorder. Performance was assessed using leave-one-subject-out cross-validation (LOSO-CV), with balanced accuracy as the primary metric. Comparative analyses were conducted using CatBoost (Yandex) and multilayer perceptron (MLP) models without pretraining. Feature importance was assessed using Shapley Additive Explanations (SHAP) values, and associations between key digital features and clinical scales were analyzed.

**Results:**

Integration of active and passive data outperformed single-modality models, achieving mean balanced accuracies of 0.71 (0.03) for SDQ-high risk, 0.67 (0.04) for insomnia, 0.77 (0.03) for suicidal ideation, and 0.70 (0.03) for eating disorder. The contrastive learning approach improved representation stability and predictive robustness. SHAP analysis highlighted clinically relevant features, such as negative thinking and location entropy, underscoring the complementary value of combining subjective and objective data. Correlation analyses confirmed meaningful associations between key digital features and mental health outcomes. Performance in an independent external validation cohort (n=45) achieved balanced accuracies of 0.63‐0.72 across outcomes, suggesting generalizability to new settings.

**Conclusions:**

This study demonstrates the feasibility and utility of smartphone-based digital phenotyping for predicting mental health risks in nonclinical, school-going adolescents. By integrating active and passive data with advanced machine modeling techniques, this approach shows promise for early detection and scalable intervention strategies in community settings.

## Introduction

Children and young people are particularly vulnerable to mental health problems due to critical developmental changes in emotion, behavior, and cognition [1], with over 75% of mental disorders emerging before the age of 25 years [2]. The World Health Organization estimates that one in 7 adolescents aged 10‐19 years at the time of this writing lives with a diagnosable mental disorder [3].

Adolescent mental health symptoms are typically classified as internalizing (eg, anxiety, depression, and suicidal thoughts) or externalizing (eg, aggression and impulsivity) [4,5]. These symptoms can impair social, academic, and interpersonal functioning, and if left unaddressed, may lead to long-term psychiatric disorders [6]. Anxiety, depression, and eating disorders are among the most prevalent conditions, with sleep problems often acting as both a symptom and a risk factor [7,8]. Among the most severe manifestations of internalizing psychopathology is suicidal ideation, a particularly serious concern during adolescence; when unaddressed, it may progress to suicide, which ranks as the third leading cause of death globally among individuals aged 15-29 years [3]. Yet only 18%‐34% of young people with significant symptoms seek professional help [9]. This critical gap in receiving care underscores an urgent need for scalable, accessible, and youth-friendly solutions in mental health care.

Given the central role of schools in adolescents’ daily lives, digital health interventions in schools offer a promising, cost-effective, and scalable way to support mental well-being in children and young people [10,11]. The proliferation of smartphones has enabled a new class of digital mental health interventions that leverage their unique data collection capabilities [12-14]. Smartphones can gather active data (subjective self-reports of behaviors and experiences) and passive data (sensor-based measures such as GPS, step count, and ambient light). These behavioral markers, collectively termed “digital phenotyping,” reveal dynamic interactions between individuals and their environments [15-17].

Active data capture internal states such as mood, sleep quality, or loneliness, but it depends on user engagement and is vulnerable to recall bias. In contrast, passive data offer continuous, objective insights into daily routines, capturing behavioral patterns that may reflect underlying mental health states. For instance, lower location entropy and reduced movement are associated with depression [18], fewer steps with lower mood [19,20], and variations in ambient light with circadian disruption [21]. However, passive data alone may miss crucial psychological context and can be challenging to interpret in isolation [22].

Although several studies [23-41] have explored either active or passive data for mental health monitoring, few have examined their integration, particularly in community-based adolescent populations. Combining these modalities provides a more comprehensive view of mental health, connecting how young people feel with how they behave in real-world settings [42]. This fusion can enhance model robustness, uncover hidden patterns, and address mismatches between self-report and behavior. For example, a young person may report feeling fine (active data) while showing signs of social withdrawal or sleep disruption (passive data), a critical challenge for unimodal approaches. Multimodal approaches are better suited to detecting such discrepancies, enabling earlier and more nuanced detection in nonclinical settings [42,43].

The complexity of digital phenotyping data, such as its high dimensionality, multimodal nature, and nonlinear interaction patterns, poses challenges for traditional statistical approaches, which rely on strong parametric assumptions and may require extensive a priori feature engineering. In contrast, machine learning (ML) can model complex, heterogeneous data and automatically learn nonlinear latent patterns without predefined hypotheses [44,45]. This makes ML particularly well-suited to adolescent mental health prediction using digital data, where subtle behavioral signals may be distributed across multiple features and modalities [17].

Despite growing interest in applying ML to digital mental health [23-41], existing studies often present one or more limitations in the context of adolescent-focused research: (1) they are primarily conducted in adult populations, limiting relevance to younger age groups such as adolescents; (2) they typically involve clinical samples, reducing generalizability to community-based or non–help-seeking populations; (3) they focus on a single condition (eg, depression or anxiety); and (4) they rely on either active or passive data, but not both. These limitations constrain the generalizability, robustness, and real-world utility of current approaches for children and young people.

This study advances the field in several ways. First, it uses a nonclinical adolescent sample, enabling evaluation in a real-world prevention context, which is critical for early detection and scalable screening. Second, it investigates 4 distinct mental health outcomes (internalizing and externalizing difficulties, insomnia, eating disorder risk, and suicidal ideation) rather than focusing on a single condition. Third, it integrates both subjective (active) and objective (passive) smartphone data to develop multimodal predictive models that capture patterns that may be missed when using either modality alone. Finally, it uses a contrastive learning framework, a novel ML technique for learning stable behavioral representations, enhancing generalizability.

Specifically, this study addresses the following research questions: (1) Can integrating active and passive smartphone data improve the prediction of mental health risks in a nonclinical adolescent population using ML? (2) How well does this multimodal approach generalize across different mental health outcomes? (3) Which digital features are most relevant to predicting specific mental health outcomes?

## Methods

### Recruitment and Data Collection

Participants were recruited from secondary schools in northwest London between November 2022 and July 2023. We contacted schools via email and followed up via telephone. Three schools that expressed interest in taking part in our study were recruited. The inclusion criteria were young people aged 14‐18 years attending 10-13 years with a sufficient level of English to respond to the study instrument and use the app and who had access to an iOS- or Android-compatible smartphone.

Students initially completed an online survey accessed via a Qualtrics link included in the promotional materials. This survey began with the Strengths and Difficulties Questionnaire (SDQ) [46], a screening tool whose predictions have been largely consistent with clinical diagnoses with good levels of internal consistency and test-retest stability. To detect eating disorders, we included the Eating Disorder-15 Questionnaire (ED‐15), which has been described as a valuable tool to assess eating disorder psychopathology in young individuals quickly [47]. We excluded the compensatory behaviors section to simplify the data collection process. Its ability to detect changes early in treatment means that it could be used as a routine outcome measure within therapeutic contexts. We also incorporated a question from the Patient Health Questionnaire version 9 (PHQ-9) [48], which is validated for young people, to identify suicidal ideation [49]. Finally, we used the Sleep Condition Indicator (SCI), a brief scale to evaluate insomnia disorder in everyday clinical practice [50,51].

Upon completing the online survey, participants received a link to download the Mindcraft app (Brain and Behaviour Lab) [52] from the App Store or Play Store, along with a unique login. Participants were asked to use the app for at least 2 weeks. The Mindcraft app is a user-friendly mobile app designed to collect self-reported well-being updates (active data) and phone sensor data (passive data). Participants set their data-sharing preferences during onboarding and can adjust them at any time through the app’s settings. Detailed technical specifications of the Mindcraft app are available in the reference [52].

### Active and Passive Data Features

Once participants began using the app, we gathered active data and 8 categories of raw passive data sourced from phone sensors and usage metrics. Active data responses (eg, mood, sleep quality, and loneliness), scored on a scale of 1-7, were directly incorporated as features for the ML model.

Passive sensing data were collected via the Mindcraft mobile app, which continuously monitored phone-based sensors and system events in the background. Sensor sampling frequencies were set to balance data resolution with device energy efficiency. All sensors except the location sensor were collected at 15-minute intervals. GPS location data were triggered by significant location changes (as determined by the operating system). From the passive data, we engineered 92 distinct features (Table 1). Location-derived features such as total distance, radius of gyration, and maximum distance from the day’s center of mass were derived from GPS logs using Haversine distance metrics and filtered for spurious location jumps. Features were aggregated over 2 time windows, the full 24-hour day and the nighttime period, defined as 10 PM to 6 AM.

A complete list of all active and passive features, along with descriptions and correlations with mental health scales, is provided in MultimediaAppendices 1  2, respectively. We highlight the key active and passive data features most strongly associated with each mental health outcome (the union of the top 3 per modality per outcome, ranked by absolute Spearman correlation) in Table 2.

Passive features that were unavailable due to user permission settings or OS-level constraints were set to a sentinel value of –1, indicating “sensor unavailable.” This allowed the model to learn platform- or user-specific absence patterns without biasing distributions. All continuous features were *z* score normalized using training set means and SDs. Binary indicator features were kept in their original form.

**Table 1.** Summary of features engineered from passive data sensors.

Passive sensor	Number of features	Feature list
Ambient light (Android only)	8	Total, mean, median, and SD of ambient light reading in the day; total, mean, median, and SD of ambient light reading during night hours
App usage (Android only)	36	Total app usage count; unique apps; total, mean, and median time usage in the day; total app usage count; unique apps; total, mean, and median time usage during the night hours; total time in app categories of camera, communication, entertainment, gaming, physical health, mental health, Mindcraft, news, productivity, and social media; percentage time in app categories of camera, communication, entertainment, gaming, physical health, mental health, Mindcraft, news, productivity, and social media
Background noise level (Android only)	10	Total, median, mean, maximum, and SD of background noise levels in the day; total, median, mean, maximum, and SD of background noise levels during night hours
Battery	8	Min, maximum, mean, and median of battery level; number of charges per day; mean battery use per hour; time below 20 percent; nighttime usage count
Location	15	Mean latitude; mean longitude; total distance traveled in a day; location count; maximum distance from home; mean distance from home; median distance from home; nighttime movement; radius of gyration; SD of latitude; SD of longitude; location entropy; time spent at home
Mindcraft usage	3	First hour of use; last hour of use; nighttime usage;
Screen brightness (iOS only)	8	Total, mean, median, SD of screen brightness sensor reading in the day; total, mean, median, SD of screen brightness sensor reading during night hours
Step count	4	Daily step count; is daily step count greater than 5000 steps or 7500 steps or 10,000 steps?

**Table 2.** Key active data and passive data features and their Spearman correlations with mental health outcomes (Strengths and Difficulties Questionnaire [SDQ], Sleep Condition Indicator [SCI], Eating Disorder-15 Questionnaire [ED-15], and suicidal ideation).

Feature type	Feature	SDQ^a^	SCI^b^	Suicidal ideation	ED-15^c^
Active	Loneliness - “How lonely are you feeling today?”	0.48^d^	–0.42^d^	0.46^d^	0.31^d^
Active	Negative thinking - “How negative do you think today?”	0.48^d^	–0.47^d^	0.57^d^	0.46^d^
Active	Racing thoughts - “Are you experiencing racing thoughts today?”	0.45^d^	–0.44^d^	0.52^d^	0.48^d^
Active	Sleep quality - “How did you sleep last night?”	–0.37^d^	0.44^d^	–0.34^d^	–0.20^d^
Active	Self-care - “How is your self-care today?”	–0.37^d^	0.33^d^	–0.24^d^	–0.42^d^
Passive	Max background noise levels over the full day	–0.49^e^	0.36	–0.39	–0.1
Passive	Mean latitude of GPS samples over the full day	0.38^f^	–0.23	0.44^e^	–0.03
Passive	Number of entertainment app usage events over the full day	0.37^e^	–0.37^f^	0.51^d^	0.41^e^
Passive	Median ambient light at night	0.06	–0.39^e^	0.14	0.27
Passive	Mean longitude of GPS samples over the full day	0.19	–0.38^f^	0.25	–0.01
Passive	SD of background noise levels over the full day	–0.43	0.36	–0.63^d^	–0.31
Passive	Median app session duration over the full day	0.37^f^	–0.19	0.44^d^	0.19
Passive	Mean background noise level at night	0.2	–0.23	0.42	0.46^f^
Passive	Total number of app usage events at night	0.09	–0.06	0.27	0.39^e^

a SDQ: Strengths and Difficulties Questionnaire.

b SCI: Sleep Condition Indicator.

c ED-15: Eating Disorder-15 Questionnaire.

d *P*<.001.

e *P*<.01.

f *P*<.05.

To reduce day-to-day variability and enhance the stability of daily feature measurements, we computed the cumulative median of each feature for every participant, that is, the median of all values up to each day. This approach progressively aggregates behavioral signals over time, dampening the influence of short-lived anomalies (eg, a one-day spike due to sensor glitches or atypical behavior, which are common in smartphone data) while preserving sustained trends. Multimedia Appendix 3 illustrates how the cumulative median stabilizes noisy input without suppressing genuine behavioral shifts, such as a consistent drop in activity due to worsening mood. In preliminary analyzes, using the same model architecture, hyperparameters, and evaluation protocol, we compared models trained on raw daily features versus those trained on cumulative median-aggregated features. We found that the latter consistently improved balanced accuracy across all outcomes (Multimedia Appendix 4). We further compared the cumulative median and the cumulative mean aggregation. While both methods yielded comparable predictive accuracy, we selected the cumulative median as the primary aggregation function due to its superior robustness to outliers, a common artifact in passive mobile sensing, thereby ensuring greater stability in user representation.

Using the engineered features, we developed an ML model for each of the 4 mental health outcomes, namely SDQ risk, insomnia, suicidal ideation, and eating disorders. We used 3 distinct feature sets, including active data, passive data, and a combination of both. This design enabled us to assess the predictive strength of each feature set individually and in combination, allowing us to systematically quantify each modality’s contribution to prediction performance across mental health outcomes.

Participants were classified as high-risk or low-risk for each outcome using validated thresholds specific to each mental health measure, framing the prediction task as a binary classification problem. Each scale’s total score range is as follows: SDQ score 0‐40, SCI 0‐32, ED-15 0‐6, and suicidal ideation 0‐4 (based on response frequency to the question, “Over the last two weeks, how often have you been bothered by thoughts that you would be better off dead or of hurting yourself in some way?”). High-risk classifications were defined as follows: for SDQ, a self-reported score of ≥16 [53]; for insomnia, if their SCI score was ≤16 [51]; for suicidal ideation, if they responded at least once to the question regarding frequency of suicidal thoughts over the last 2 weeks, and for eating disorders, an ED-15 total score exceeded 2.69, which corresponds to the mean plus one SD in a nonclinical population [54]. The proportions of participants classified as high-risk for each mental health outcome are summarized in Table 3.

**Table 3.** Demographics and mental health measures of the study population (N=103).

Variables	Values
Sex (Female), n (%)	73 (70.9)
Age (years), mean (SD)	16.1 (1)
Strengths and Difficulties Questionnaire (SDQ) score, mean (SD)	12.8 (6.2)
High-risk SDQ category (SDQ score≥16), n (%)	31 (30.1)
Eating Disorder (ED-15) scale, mean (SD)	2.2 (1.8)
High-risk eating disorder category (ED-15 score≥2.7), n (%)	38 (36.9)
Sleep Condition Indicator (SCI) score, mean (SD)	19.9 (7.8)
High-risk insomnia category (SCI score<17), n (%)	34 (33)
“Over the last two weeks, how often have you been bothered by thoughts that you would be better off dead or of hurting yourself in some way?” mean (SD)	0.6 (0.9)
High-risk suicidal ideation category (≥1), n (%)	38 (36.9)

### ML Workflow and Model Development

**Figure 1.**
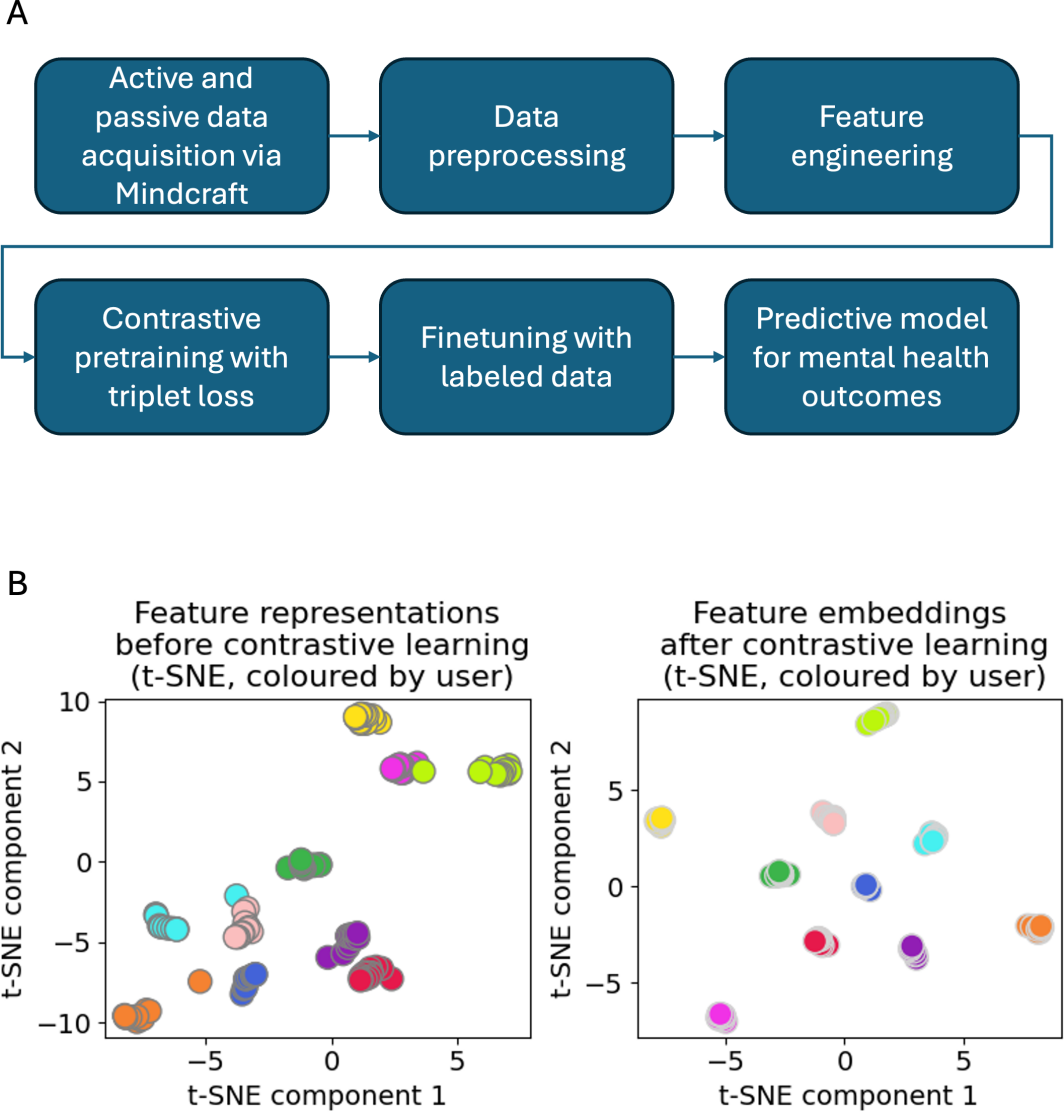
Overview of the machine learning (ML) framework for predicting adolescent mental health outcomes using smartphone-based digital phenotyping. (A) Workflow of the ML pipeline, from data acquisition to mental health outcome prediction, incorporating contrastive pretraining with triplet loss and fine-tuning. (B) t-SNE visualization of feature embeddings for a sample of 10 test users before (left) and after (right) contrastive pretraining, showing enhanced user-specific clustering following pretraining. t-SNE: t-distributed Stochastic Neighbor Embedding.

Figure 1A outlines our ML pipeline, starting with active and passive data collection via the Mindcraft app. The data were preprocessed and engineered to create a comprehensive feature set, which was subjected to a pretraining phase with contrastive learning using triplet margin loss. This pretraining step clustered user-specific features from different days, minimizing day-to-day variability and preserving individual behavioral patterns. The resulting stable embeddings were fine-tuned on labeled data in a supervised setting to predict mental health outcomes. This end-to-end pipeline, combining pretraining and fine-tuning, enabled the development of a predictive model evaluated using balanced accuracy and additional metrics.

### Contrastive Pretraining Model Architecture and Training

To accurately predict mental health risks, it is essential to distinguish between a user’s stable behavioral patterns and random daily fluctuations. Raw smartphone data can vary significantly day to day due to external factors (such as school holidays) unrelated to mental health. To address this, we used an ML technique known as contrastive learning with triplet margin loss. In simple terms, this technique teaches the model to recognize a user’s unique digital fingerprint. It does so by examining 3 data points (a “triplet”) simultaneously:

- Anchor: data from a specific user on a particular day (eg, User A, Monday).
- Positive: data from the same user on a different day (eg, User A, Thursday).
- Negative: data from a different user entirely (eg, User B, Monday).

The model is trained to minimize the distance between the Anchor and the Positive (pulling them together in mathematical space) while maximizing the distance between the Anchor and the Negative (pushing them apart). Repeating this process across thousands of triplets many times helps the model learn to ignore irrelevant daily noise and form a stable, underlying behavioral fingerprint unique to that user. These stable representations are then used for the subsequent supervised prediction of mental health risk. A simplified visual explanation of this idea is provided in Figure 1B, which helps understand why contrastive pretraining is valuable in datasets with naturally high daily variability, such as smartphone-based behavioral data.

We implemented a custom contrastive learning pipeline in PyTorch (Meta AI), based on established principles of triplet-based metric learning [55,56], with the objective of learning robust, user-specific feature embeddings that remain consistent across days, thereby reducing intrasubject variability while maximizing intersubject separability. For this pretraining phase, we selected triplet margin loss over other contrastive objectives (eg, InfoNCE) due to its suitability for instance-level metric learning, where the aim is to ensure that embeddings from the same user are closer in the latent space than those from different users. Compared to InfoNCE, triplet loss is more stable with moderate mini-batch sizes and is better suited to high-dimensional tabular data [55,57].

Triplets were constructed without using mental health outcome labels, as the pretraining phase was fully self-supervised. A user was first randomly selected from the training set, and a single day from their data was sampled as the Anchor. The Positive sample was randomly chosen from a different day for the same user, while the Negative was drawn from a random day of a different user. The triplet loss requires that an Anchor data point is closer to a Positive data point than it is to a Negative data point, by at least a specified margin m.

The contrastive learning model consisted of a 2-layer multilayer perceptron (MLP) encoder, which mapped input features to a latent embedding space, followed by a 2-layer MLP projection head. The projection head was trained using the triplet loss, allowing the encoder to retain generalizable behavioral representations while the projection space focused on the contrastive objective [58]. The Adam optimizer was used for pretraining with a fixed learning rate of 1×10^−3^. The model was pretrained for 3 epochs with a batch size of 256 triplets. The margin m was set to 1.0 in our experiments.

### Supervised Fine-Tuning Model Architecture and Training

Following contrastive pretraining, the learned encoder was used as a fixed base for the downstream classification tasks. Its weights were frozen to preserve the stable user-specific embeddings. A new 2-layer MLP classification head was added on top of the frozen encoder to perform binary classification for each mental health outcome. The fine-tuning model was trained using the Adam optimizer with a learning rate of 1×10^–^³ for 20 epochs and a batch size of 1024. Binary cross-entropy with logits loss was used as the training objective, with class weights applied to account for imbalance. These weights were calculated based on the inverse frequency of each class within the training set of each cross-validation fold.

Key architectural hyperparameters (eg, number of layers, dimensions of embeddings, and projections) and training hyperparameters (eg, learning rate, batch size, triplet margin, and number of epochs) were selected based on common practice in contrastive learning and refined through exploratory tuning on a validation split of the training data within the first cross-validation fold. No test user data from any fold was used during hyperparameter selection. All selected hyperparameters were then fixed across folds to ensure methodological consistency and reproducibility and to prevent data leakage.

To demonstrate the effect of contrastive pretraining, Figure 1B shows t-distributed Stochastic Neighbor Embedding visualizations of feature embeddings for a sample of 10 test users. Before pretraining (left plot), user-specific data points were scattered with minimal clustering, reflecting high day-to-day variability. After applying contrastive learning with triplet loss (right plot), data points from the same user formed tighter clusters, indicating enhanced user-specific feature stability.

### Evaluation and Benchmarking

Day-wise prediction probabilities were averaged for each test user to obtain a single, user-level prediction. Model performance was evaluated using balanced accuracy as the primary metric, along with other relevant metrics such as area under the receiver operating characteristic curve (AUC) and *F*_1_-score, to assess classification outcomes comprehensively. For interpretability, Shapley additive explanations (SHAP) values [59] were computed for test folds using DeepExplainer [60], offering insight into the contributions of specific features to classification outcomes.

We validated the model’s performance using leave-one-subject-out cross-validation (LOSO-CV), where data from all but one user were used for training and validation, with the excluded user’s data serving as the test set. This approach ensures the model’s generalizability to new individuals, closely simulating real-world applications where accurate predictions for unseen users are critical.

To benchmark our model, we compared it with a CatBoost (Yandex) classifier [61] and an MLP network that had a similar number of parameters but was trained without pretraining. Both benchmarks used class-weight balancing to address the class imbalance in the dataset. CatBoost was selected for its strong performance on tabular data and its built-in capability to handle class imbalance [62], making it well-suited for datasets like ours. These comparisons isolated the effect of contrastive pretraining on model performance.

### External Validation on an Independent Cohort

We collected data from an additional cohort of 90 adolescents across 5 new secondary schools in London. To create an independent external validation set, we randomly split this new cohort into 2 halves. One half (n=45) was added to the original dataset to form an expanded training set, while the remaining half (n=45) served as a fully held-out external validation cohort. Importantly, no model architecture, hyperparameters, preprocessing, or feature engineering steps were modified after adding the new data; the complete pipeline, including contrastive pretraining and supervised fine-tuning, remained fixed. This design allowed us to assess generalizability to users from different schools, collected at a later time point, with different demographic characteristics and smartphone-sensor enabling patterns. Model performance on this external cohort was evaluated using balanced accuracy following the same user-level prediction protocol used in the original LOSO-CV analysis.

### Ethical Considerations

Ethics approval for the study was granted by the Imperial College London Research Ethics Committee (reference number: ICREC 20IC6132). All procedures complied with relevant national and institutional ethical standards and with the Declaration of Helsinki. Informed consent was obtained digitally from all participants at the start of the survey; for participants younger than 16 years, parental consent was obtained before participation. Participants could withdraw from the study at any time before the start of data analysis. Study-specific identification numbers were used to maintain participant anonymity, and data handling complied with the General Data Protection Regulation (GDPR) for health and care research. Participation was voluntary and uncompensated. Multimedia Appendix 5 provides a CONSORT (Consolidated Standards of Reporting Trials)-style flow diagram outlining the subject inclusion process from the initial 205 survey respondents.

## Results

### Recruitment and App Usage

**Figure 2.**
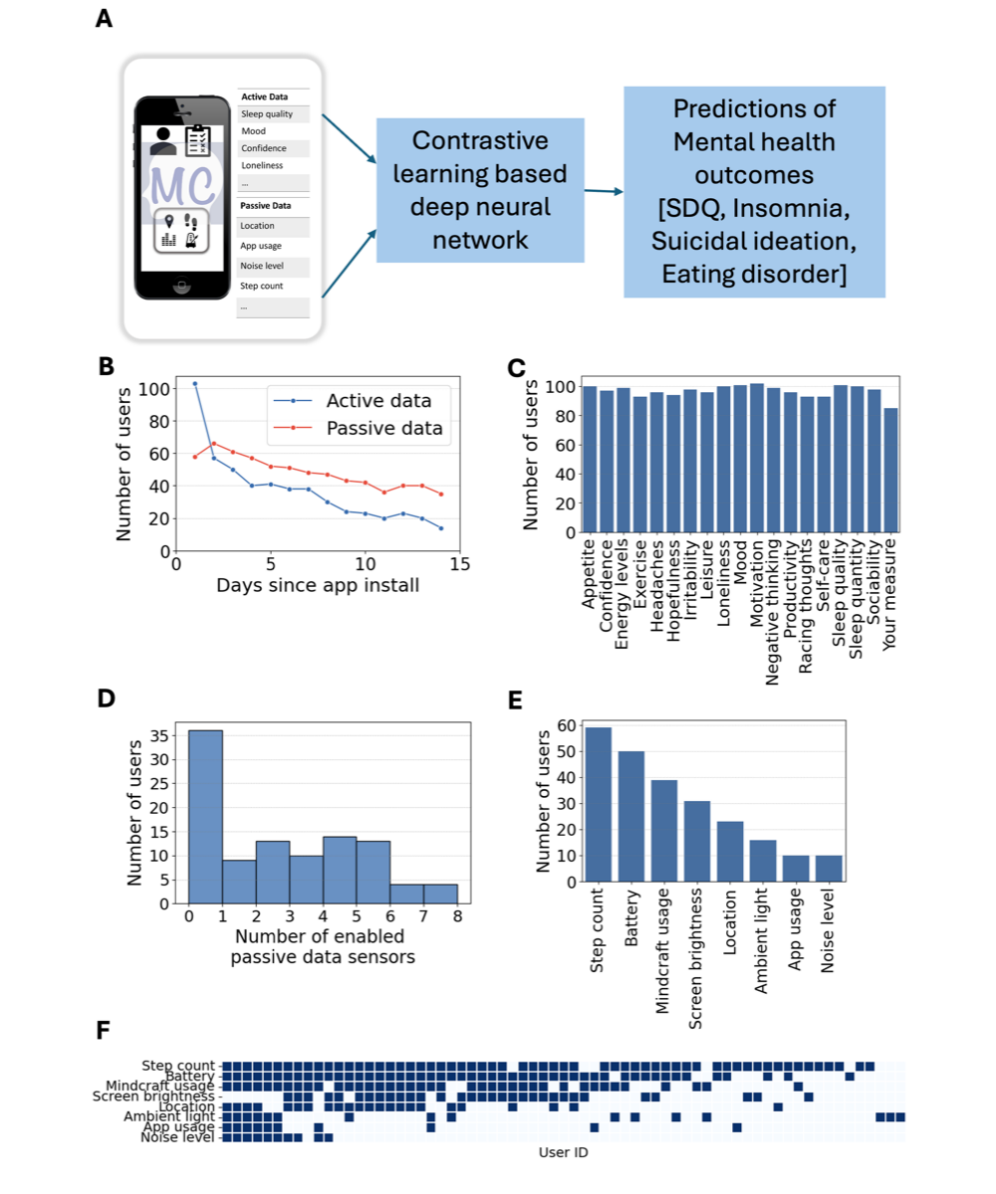
Study overview and participant engagement with active and passive data collection using the Mindcraft app. (A) Conceptual overview of the study. (B) User engagement trends for active and passive data over the 14-day period. (C) User participation across active data questions. (D) Distribution of enabled passive sensors among users. (E) User participation across different passive sensor types. (F) Heatmap of passive data completeness by user and sensor type. SDQ: Strengths and Difficulties Questionnaire.

Figure 2A provides a conceptual overview of the study, demonstrating how active data (eg, sleep quality, mood, and loneliness) and passive data (eg, location, app usage, and noise levels) collected via the Mindcraft app are integrated into a contrastive learning-based deep neural network to predict mental health outcomes, including SDQ risk, insomnia, suicidal ideation, and eating disorders.

A total of 103 students from 3 London schools downloaded and installed the Mindcraft app. The average age was 16.1 years (SD 1), with 71% identifying as female, 25% as male, and 4% as other or nonbinary. The skew in gender distribution is partially due to one of the participating schools being girls-only. Of the participants, 78 used the app on iPhones, and 25 used Android phones. Table 3 provides demographic information and mental health outcome scores, and Multimedia Appendix 6 illustrates the distribution of the different mental health measures across our study population.

Participants contributed active data via self-reported measures and passive data through smartphone sensors. Active data included daily ratings of mental well-being measures such as sleep quality, mood, confidence, and loneliness on a 1‐7 scale. Passive data comprised data from phone sensors like location, app usage, ambient noise, and step count. Figure 2B shows user engagement patterns over the 14-day study period. Initial engagement was high, with all participants contributing at baseline. However, active data engagement declined more rapidly than passive data, with 14 users contributing active data and 36 users contributing passive data on day 14.

Engagement with active data measures (Figure 2C) remained consistent across users, with slight variations reflecting individual preferences. In contrast, passive data collection exhibited substantial variability (Figure 2D). While 36 users opted not to enable any sensors, others enabled multiple categories. The most frequently enabled sensors were step count and battery usage, followed by Mindcraft usage and screen brightness (Figure 2E). The heatmap visualization of passive data coverage by users and sensor type (Figure 2F) underscores substantial interuser variability, with some users providing comprehensive coverage across multiple sensors and others contributing sporadically.

### Exploratory Analysis of Active and Passive Data Features

**Figure 3.**
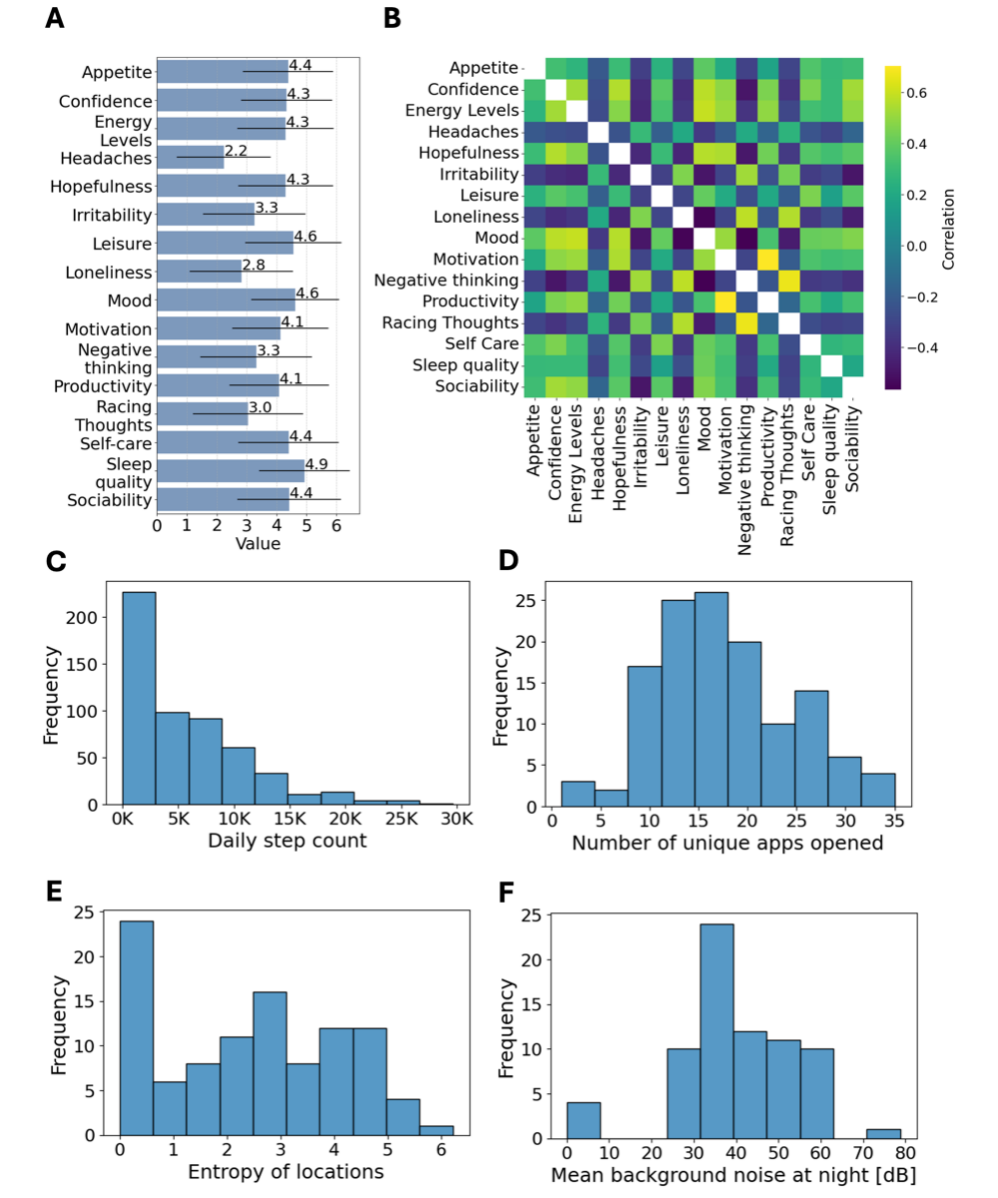
Descriptive statistics of active and passive digital phenotyping features. (A) Distribution of responses across active data features. (B) Correlation heatmap of active data features. (C) Frequency distributions of passive data features: daily step count. (D) Number of unique apps opened per day. (E) Location entropy reflecting movement variability. (F) Mean background noise levels at night.

Figure 3 provides an overview of descriptive statistics and correlations among active and passive data features collected from users. Figure 3A illustrates the distribution of self-reported active data features on a scale of 1-7. Positive indicators, such as mood, motivation, and confidence, had higher mean values than negative indicators, such as negative thinking, racing thoughts, and irritability.

The correlation heatmap (Figure 3B) highlights relationships among active data features. A fully annotated, high-resolution version of this heatmap is provided in Multimedia Appendix 7. The strongest correlation (*r*=0.7) was observed between motivation and productivity, followed by a strong association between negative thinking and racing thoughts (*r*=0.66). Positive correlations were also observed between 2 well-being indicators, energy levels and mood (*r*=0.58), and between 2 distress indicators, loneliness and negative thinking (*r*=0.57). Conversely, negative correlations were seen, such as between mood and negative thinking (*r*=–0.56) and irritability and sociability (*r*=–0.49).

Figures 3C-3F illustrate 4 of the 92 engineered passive data features. The distribution of daily step counts (Figure 3C) is right-skewed, with most users taking fewer than 10,000 steps per day. Figure 3D shows the frequency of unique apps opened daily, peaking at 15‐20 apps, indicating varying levels of mobile engagement. The entropy of locations visited (Figure 3E) reflects movement variability, with higher values suggesting diverse activity patterns. Finally, Figure 3F highlights the distribution of mean background noise levels at night, clustering between 30 and 50 decibels.

### Associations Between Active and Passive Features and Clinical Outcomes

To assess the alignment between digital phenotyping features and clinical mental health symptoms, we examined Spearman correlations between active and passive features and continuous scores on the SDQ, SCI, ED-15, and suicidal ideation frequency. Among active features, negative thinking consistently showed the strongest associations across all outcomes (ρ=0.48 with SDQ, ρ=–0.47 with SCI, ρ=0.57 with suicidal ideation, ρ=0.48 with ED-15, all *P*<.001). Higher levels of racing thoughts and loneliness were associated with more severe mental health symptoms, whereas greater confidence and hopefulness were linked to reduced risk. Key passive features also demonstrated moderate associations with outcomes. For instance, greater entertainment app usage consistently showed associations across all outcomes (ρ=0.37 with SDQ, ρ=–0.37 with SCI, ρ=0.51 with suicidal ideation, ρ=0.41 with ED-15, all *P*<.05). Nighttime ambient light exposure and location variability (latitude and longitude) were also relevant across multiple outcomes, particularly for insomnia and eating disorder symptoms. Key active and passive features, along with their correlations with each outcome, are summarized in Table 2. MultimediaAppendices 1  2 list detailed descriptions and Spearman correlations for all active and passive features used in the predictive modeling pipeline, offering an overview of their associations with SDQ, SCI, ED-15, and suicidal ideation.

To further illustrate the discriminative capacity of key digital phenotyping features, we compared the top 5 active and passive features between high- and low-risk groups for each mental health outcome. Multimedia Appendix 8 shows the variability in these features across the 4 outcomes (SDQ, insomnia, suicidal ideation, and eating disorder). The observed group differences are consistent with the correlation-based findings and reinforce the predictive relevance of both active and passive data streams in distinguishing individuals at elevated mental health risk.

### Performance of Models Predicting Mental Health Outcomes

**Figure 4.**
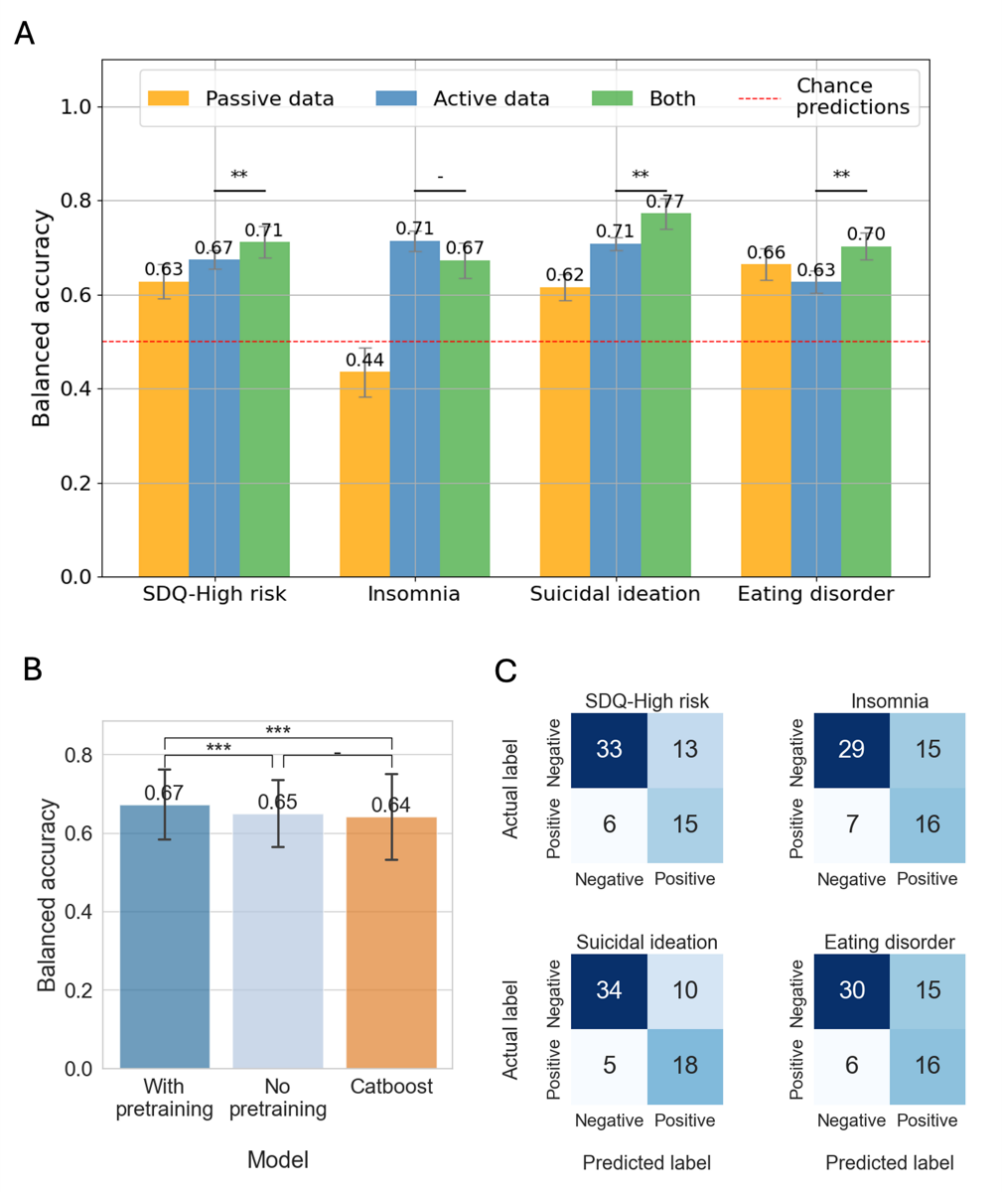
Model performance for predicting mental health outcomes using active and passive digital phenotyping data. (A) Balanced accuracy of mental health outcome predictions (SDQ-high risk, insomnia, suicidal ideation, and eating disorder) using passive, active, and combined data. (B) Comparison of balanced accuracy for models with contrastive pretraining, without pretraining, and a CatBoost model, showing the performance benefit of pretraining (*P*<.001, paired *t* test). (C) Confusion matrices for the combined data model’s predictions, showing true-positive and true-negative classifications across mental health outcomes. *: *P*<.05; **: *P*<.01; ***: *P*<.001; SDQ: Strengths and Difficulties Questionnaire.

Building on these associations, we evaluated how well ML models could predict mental health outcomes using active, passive, and combined data. Figure 4A illustrates the balanced accuracy of predictive models for the 4 mental health outcomes (SDQ-high risk, insomnia, suicidal ideation, and eating disorder) evaluated across 10 repetitions of LOSO-CV. The analysis involved 3 feature sets, including passive data, active data, and a combination of both. This evaluation was restricted to the 67 participants who provided both active and passive data to ensure a fair comparison. The red dashed line indicates chance-level performance, and statistically significant differences are denoted by asterisks (*P<.05, **P<.01, ***P<.001; Wilcoxon signed-rank test).

For SDQ-high risk, the model using passive data achieved a balanced accuracy of 0.63, while active data alone reached 0.67 (MultimediaAppendices 1  2). The combined model, leveraging both data types, achieved a significantly higher balanced accuracy of 0.71 compared to active data alone (*P*=.003, Wilcoxon signed-rank test). Similarly, the combined data model outperformed the active data alone for eating disorder predictions, with balanced accuracies of 0.70 and 0.63, respectively (*P*=.003; Wilcoxon signed-rank test). In predicting insomnia, the combined model achieved a balanced accuracy of 0.67, while passive data alone performed below the chance level (0.44). For suicidal ideation, the combined model achieved the highest balanced accuracy of 0.77, significantly outperforming both active data (0.71) and passive data (0.62; *P*=.003; Wilcoxon signed-rank test). Table 4 summarizes additional performance metrics, including the AUC, the area under the precision-recall curve, *F*_1_-scores, sensitivity, specificity, precision, and recall for each mental health outcome.

**Table 4.** Detailed performance metrics for mental health outcome predictions.

Metric	SDQ^a^-high risk, mean (SD)	Insomnia, mean (SD)	Suicidal ideation, mean (SD)	Eating disorder, mean (SD)
Balanced accuracy	0.71 (0.03)	0.67 (0.04)	0.77 (0.03)	0.70 (0.03)
AUC^b^	0.77 (0.03)	0.74 (0.02)	0.82 (0.03)	0.73 (0.02)
AUC-PR^c^	0.53 (0.04)	0.52 (0.05)	0.64 (0.05)	0.52 (0.03)
*F*1-score	0.61 (0.04)	0.59 (0.04)	0.70 (0.04)	0.61 (0.03)
F1 macro	0.69 (0.03)	0.66 (0.04)	0.76 (0.03)	0.68 (0.03)
Sensitivity	0.71 (0.06)	0.68 (0.03)	0.78 (0.05)	0.74 (0.03)
Specificity	0.71 (0.05)	0.66 (0.07)	0.77 (0.04)	0.67 (0.04)
Precision	0.53 (0.04)	0.52 (0.05)	0.64 (0.05)	0.52 (0.03)
Recall	0.71 (0.06)	0.68 (0.03)	0.78 (0.05)	0.74 (0.03)

a SDQ: Strengths and Difficulties Questionnaire.

b AUC: area under the receiver operating characteristic curve.

c AUC-PR: area under the precision-recall curve.

Figure 4B demonstrates the effectiveness of contrastive pretraining. Models with pretraining achieved the highest balanced accuracy (0.67), significantly outperforming both the model without pretraining (0.65; *P*<.001; paired 2-tailed *t* test) and the CatBoost model (0.64; *P*<.001; paired 2-tailed *t* test).

Figure 4C presents the confusion matrices for the combined data models. For SDQ-high risk, the model correctly identified 33 negatives and 15 positives, with 6 false negatives and 13 false positives. The model had higher misclassification rates for insomnia, with 7 false negatives and 15 false positives. In predicting suicidal ideation, the model demonstrated strong performance, correctly classifying 34 negatives and 18 positives, with only 5 false negatives and 10 false positives. Similarly, for eating disorders, the model accurately identified 30 negatives and 16 positives, with 6 false negatives and 15 false positives.

To assess whether the active-only model’s predictive performance generalizes across user subgroups, we compared balanced accuracy for the subset (n=67) who provided both active and passive data with that of the full cohort (n=103), which includes participants who shared only active data (Multimedia Appendix 9). The consistent results across these 2 groups indicate that the active-only model’s performance is stable and not restricted to a specific subgroup, thereby confirming that restricting the analysis to the subset of users with both active and passive data (as done in Figure 4A) has not introduced bias in model performance.

### External Validation Performance on an Independent Cohort

**Figure 5.**
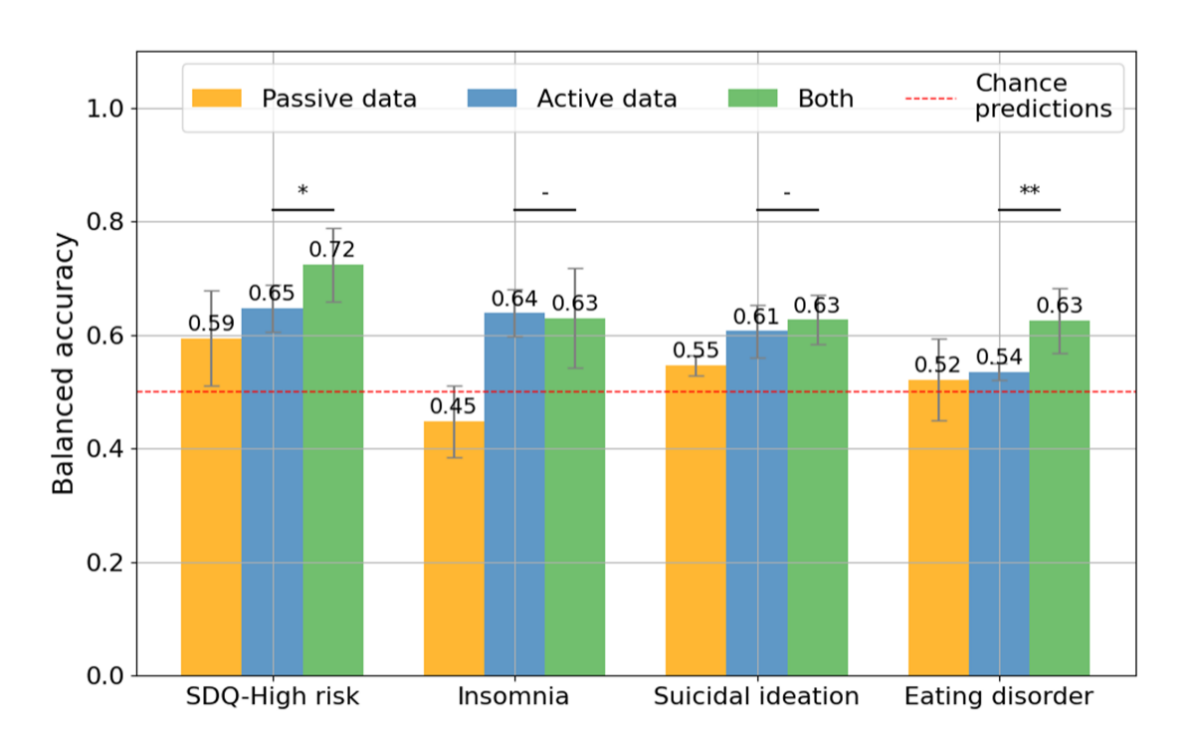
External validation performance on an independent cohort. Balanced accuracy for predicting SDQ high risk, insomnia, suicidal ideation, and eating disorder risk in an external validation sample (n=45 adolescents not used for model development). Error bars indicate SD across 10 repeated runs. *: *P*<.05; **: *P*<.01; ***: *P*<.001; SDQ: Strengths and Difficulties Questionnaire.

Model performance on the held-out external validation cohort (n=45 adolescents from 5 additional schools; none of these adolescents’ data were used for model development) showed a similar pattern to the LOSO-CV results, albeit with reduced accuracy for some outcomes (Figure 5). Figure 5 shows balanced accuracy for models trained using passive data only, active data only, and combined active and passive data; error bars indicate standard deviation across 10 repeated runs. The red dashed line indicates chance-level performance, and statistically significant differences are denoted by asterisks (*P<.05, **P<.01, ***P<.001; Wilcoxon signed-rank test). Using combined active and passive data, balanced accuracy in the external cohort was 0.72 for SDQ-high risk, 0.63 for insomnia, 0.63 for suicidal ideation, and 0.63 for eating disorder risk (vs 0.71, 0.67, 0.77, and 0.70, respectively, in the LOSO-CV analysis). Models trained using passive data only and active data only showed the same qualitative pattern, with active and combined data generally outperforming passive data alone, but with lower accuracies than in LOSO-CV, particularly for suicidal ideation and eating disorders. A full set of evaluation metrics for the combined model in the external validation sample is provided in Multimedia Appendix 10. Overall, the external validation results showed a similar performance pattern to the LOSO-CV analysis, with reduced accuracy for suicidal ideation and eating disorder.

### Model Fairness Across Gender and School Contexts

Given known differences in smartphone use and mental health between demographic groups, we examined whether model performance varied systematically by gender (Multimedia Appendix 11). Participants were grouped into female (n=46) and male and other (n=21). For the combined active and passive model, balanced accuracy was broadly comparable across gender for all 4 outcomes, with differences modest in magnitude and inconsistent in direction (eg, suicidal ideation: 0.73 vs 0.74). Error bars overlapped for all outcomes, and no subgroup was consistently disadvantaged, suggesting no evidence of systematic gender-related performance degradation in this sample.

Because individual-level socioeconomic status (SES) data were unavailable, we used school as a contextual proxy for both SES and the institutional environment. The 3 participating schools differed in selectivity, gender composition, and catchment-area deprivation: School 1 (girls-only, partially selective grammar in a mid-SES area), School 2 (mixed-gender community school in a deprived urban area), and School 3 (mixed-gender selective sixth-form college in a mid-to-higher SES area). For each school, we compared balanced accuracy for students from that school with that for students from the other 2 schools combined (Multimedia Appendix 11). Across all 4 outcomes, differences were varied in direction rather than systematically favoring or disadvantaging any single school (eg, School 1 vs others: SDQ 0.71 vs 0.73; insomnia 0.76 vs 0.59; suicidal ideation 0.68 vs 0.76; eating disorder 0.65 vs 0.71), with overlapping CIs in all cases. Overall, these analyses provide no evidence of systematic performance degradation associated with school context or school-level SES, although the study was not powered to detect more subtle or intersectional fairness effects.

### Robustness to Heterogeneous Sensor Activation

To evaluate whether missing passive-sensor streams introduced systematic bias, we stratified participants based on the number of passive sensors enabled. The median number of sensors in the dataset was 3; therefore, we compared model performance between users with ≤3 sensors enabled and those with >3 sensors enabled (Multimedia Appendix 12). Model performance was evaluated separately for each subgroup for all 4 outcomes using the combined active-and-passive model. Differences in balanced accuracy were inconsistent in direction across outcomes, indicating no evidence of systematic performance degradation for users with more missing sensor data. Notably, for suicidal ideation, the 2 subgroups performed nearly identically (0.74 vs 0.72). For eating disorder risk, the ≤3-sensor group performed better (0.80 vs 0.56), suggesting that the core predictive signals for this outcome are captured within the most commonly enabled sensors and that additional sensors may introduce noise for this specific target. These results provide no evidence that heterogeneous sensor activation introduced systematic group-level bias against users with fewer sensors. They also suggest that the combined model is reasonably robust to the observed patterns of missing passive data. However, our sample size limits the detection of more subtle fairness effects related to specific sensor combinations or intersectional subgroups.

### Predictive Accuracy Across Mental Health Risk Groups

**Figure 6.**
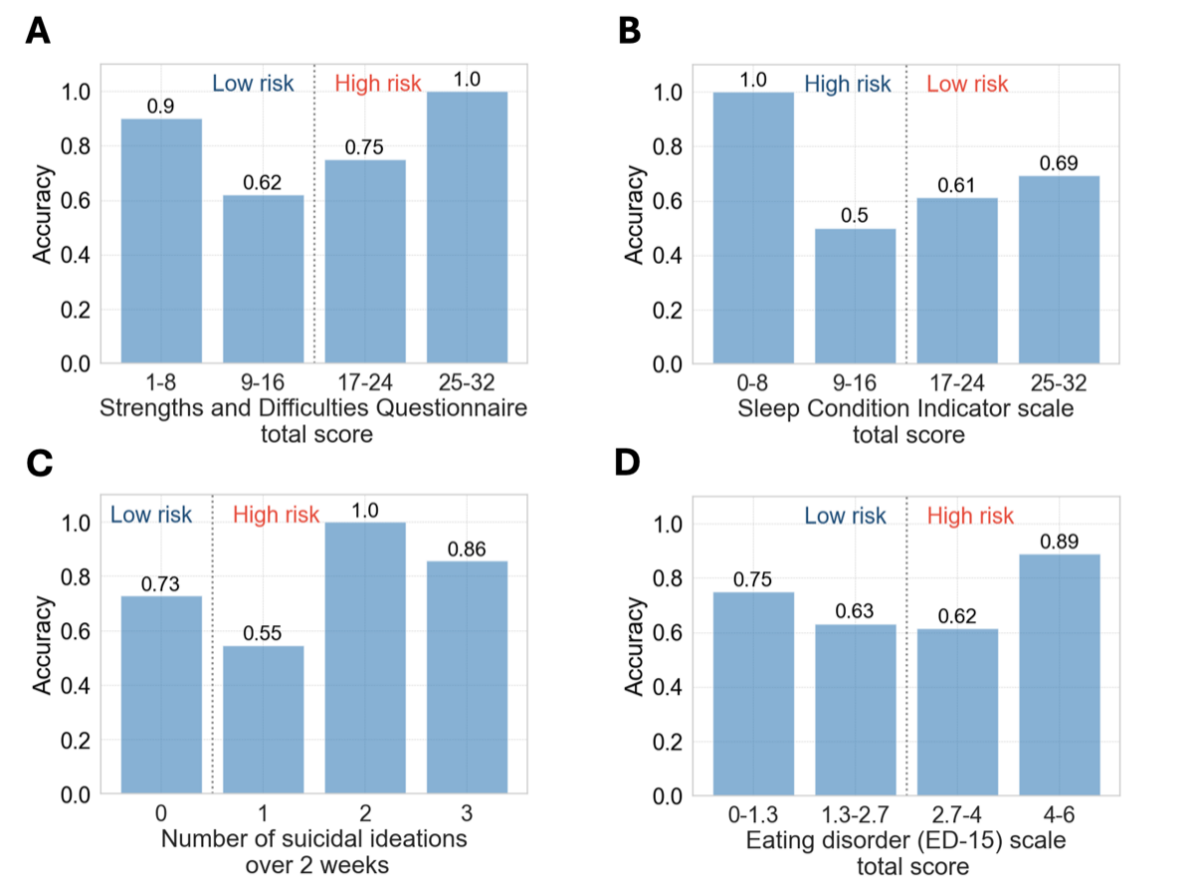
Accuracy of mental health risk prediction across different levels of (A) Strengths and Difficulties Questionnaire (SDQ) total score. (B) Sleep Condition Indicator (SCI) score. (C) Frequency of suicidal ideation thoughts. (D) Eating Disorder (ED-15) total score.

Figure 6 illustrates model accuracy in predicting mental health risks using combined active and passive data, segmented by risk levels for various mental health measures. The model performed exceptionally well at extreme risk levels, achieving near-perfect accuracy for high-risk groups (eg, SCI scores 0‐8 and EDQ scores 4‐6) and low-risk groups (eg, SDQ scores 1‐8). However, accuracy decreased significantly in ranges near thresholds (eg, SDQ scores 9‐16 and SCI scores 9‐16).

### Model Interpretability: Active and Passive Data Contributions

**Figure 7.**
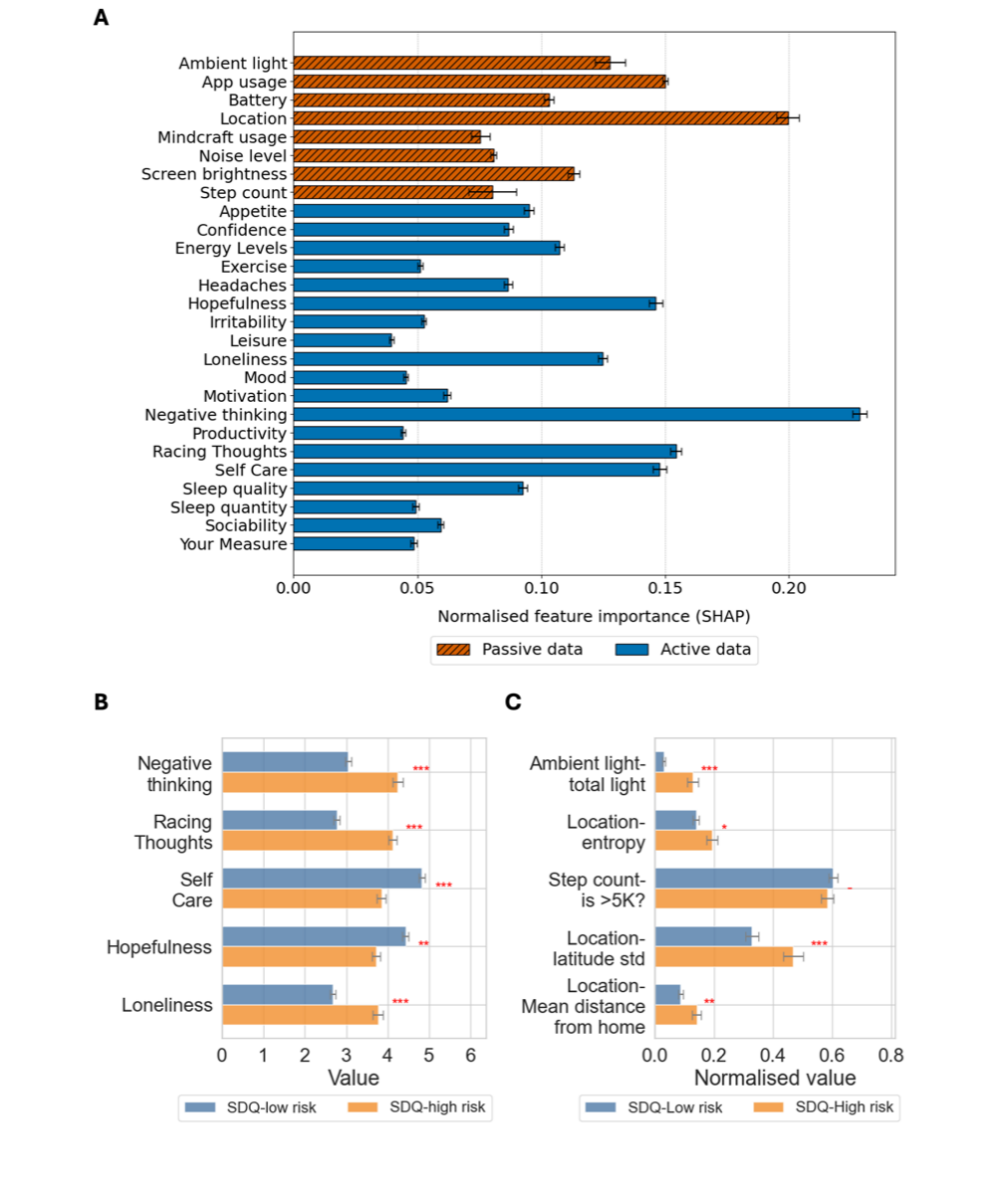
Feature importance analysis for predicting the SDQ high-risk category using both active and passive data. (A) SHAP-based feature importances, with passive data aggregated by sensor type and active data shown individually. (B) Distribution of the top five active data features across SDQ risk categories. (C) Distribution of the top five passive data features across SDQ risk categories. Statistically significant differences between low-risk and high-risk groups are indicated (*: *P*<.05, **: *P*<.01, ***: *P*<.001, *t* test). SDQ: Strengths and Difficulties Questionnaire; SHAP: Shapley Additive Explanations.

Figure 7A illustrates the feature importance calculated using SHAP values for predicting the SDQ high-risk category using a combination of both active and passive data, with passive data aggregated by sensor type and active data shown individually. The top predictors included negative thinking, location features, app usage, racing thoughts, and self-care. Cognitive and emotional indicators (eg, negative thinking and racing thoughts) ranked highest among active data features, while movement and environmental stability (eg, location entropy and step count) dominated passive data contributions. Corresponding SHAP-based feature importance plots for predicting insomnia, suicidal ideation, and eating disorders are provided in Multimedia Appendices 13-15, respectively.

The distribution of the top five active data features (negative thinking, racing thoughts, self-care, hopefulness, and loneliness) showed clear distinctions between low- and high-risk SDQ groups (Figure 7B). Negative thinking and racing thoughts were significantly higher in the high-risk group (*P*<.001; *t* test). Conversely, self-care (*P*<.001; *t* test) and hopefulness (*P*<.001; *t* test) were significantly lower. Loneliness was also notably higher in the high-risk group (*P*<.001; *t* test).

The distribution of the top 5 passive data features (ambient light, location entropy, step count, latitude SD, and mean distance from home) highlighted significant differences between risk groups (Figure 7C). High-risk individuals showed greater ambient light exposure (*P*<.001; *t* test), potentially reflecting greater exposure to light at night and sleep disruptions. They also exhibited higher location entropy (*P*=.02; *t* test) and latitude variability (*P*<.001; *t* test). Additionally, fewer high-risk individuals exceeded 5000 daily steps (*P*=.002; *t* test).

## Discussion

### Principal Findings

Our study demonstrated the effectiveness of integrating active self-reported and passive smartphone sensor data to predict adolescent mental health risks using a novel ML framework. By leveraging data collected via the Mindcraft app, we evaluated predictions across 4 critical mental health outcomes, namely SDQ-high risk, insomnia, suicidal ideation, and eating disorders. Combined models consistently outperformed unimodal approaches, achieving competitive balanced accuracies across all outcomes (eg, 0.77 for suicidal ideation and 0.71 for SDQ-high risk), even in a broad, nonclinical adolescent sample (Figure 4A). These results highlight the complementary value of passive data, which unobtrusively captures continuous behavioral patterns that enrich the subjective insights provided by active data.

Importantly, these results were obtained in a nonclinical, school-based adolescent cohort that was deliberately broad, behaviorally diverse, and not selected for help-seeking status. Such populations introduce substantial intragroup variability, natural fluctuations in engagement, and noise in both active and passive measures, making predictive modeling especially challenging. Previous digital phenotyping studies in non-clinical adolescent or student samples have reported the AUC or *F*_1_-scores typically ranging from 0.60 to 0.80 when predicting stress, depression, or anxiety using mobile sensing [35-41]. Many of these studies also involved smaller or more homogeneous samples and often focused on a single outcome. Our balanced accuracies of 0.70‐0.77 are therefore competitive, especially given that our study simultaneously addressed 4 distinct mental health outcomes in a naturalistic, non–help-seeking adolescent sample. The external validation results suggest that the learned behavioral representations generalize reasonably well to new schools and time periods, while also highlighting some loss of accuracy for the more challenging outcomes.

User engagement patterns indicated sustained utility of passive data collection, underscoring its lower participant burden and feasibility in scalable longitudinal mental health monitoring. Participants preferred less intrusive metrics, including step count, battery usage, and screen brightness, emphasizing the importance of prioritizing user-friendly data collection methods. Our innovative contrastive learning approach effectively addressed variability inherent in daily behavioral data, stabilizing user-specific feature representations. This methodological advancement yielded improved performance and increased confidence in the model’s applicability to the real world.

### Comparison With Prior Work

Previous work has largely focused on adults or clinical populations [23-40], limiting its applicability to adolescents in community settings. Even among adolescent-focused studies [25,33,34,63,64], most relied solely on passive sensing and targeted depression or anxiety, limiting the generalizability of their findings to broader, nonclinical groups.

Digital self-monitoring has a potential role in multiple stages of the clinical pathway, from prevention to clinical intervention. Our work addresses a broader range of mental health outcomes of internalizing and externalizing disorders, eating disorders, insomnia, and the presence of suicidal ideation in a nonclinical, non–help-seeking school-going adolescent population. MacLeod et al [63], the closest study to ours, included younger adolescents from clinical and nonclinical settings but relied solely on passive sensing. To our knowledge, this study is the first to use ML to accurately predict mental health risk across a broad range of outcomes in low- and higher-risk school-going adolescents, using a combination of active and passive data in a general, nonclinical population.

To interpret our results in light of previous literature, we discuss the findings for each mental health outcome in our study individually below.

#### SDQ

SHAP analysis highlighted key passive features (Figure 7C), including lower step count, increased location entropy, and elevated ambient light exposure, which were linked to behavioral and environmental patterns associated with mental health risk, consistent with prior studies on physical inactivity, disrupted routines, and light exposure [18-21]. Fewer high-risk individuals exceeded 5000 daily steps, reinforcing associations with sedentary behavior [19,20]. Location entropy, capturing variability in movement, may signal a lack of daily structure [18], while ambient light patterns could reflect disturbed circadian rhythms [21]. In parallel, active features such as negative thinking, racing thoughts, and poor self-care also ranked highly (Figure 7B) and aligned with literature on internalizing and externalizing symptomatology [65-67].

#### Insomnia

Predictions were driven by active features such as self-care, negative thinking, and appetite, and by passive measures such as app usage hours, nighttime movement, and ambient light (Multimedia Appendix 13). This aligns with earlier research highlighting subjective perceptions, such as increased negative thinking, loneliness, and decreased self-care and hopefulness, as critical factors in sleep disturbances [68,69]. The significance of ambient light exposure further supports evidence linking circadian disruptions to poor sleep quality in adolescents [70,71].

#### Suicidal Ideation

Key active features (Multimedia Appendix 10), including negative thinking, loneliness, and reduced hopefulness, reflect core cognitive-affective vulnerabilities and are consistent with prior evidence linking these traits to elevated suicide risk in adolescents [72,73]. Passive features (Multimedia Appendix 13) such as screen brightness variability and late-night Mindcraft app usage may indicate disrupted circadian rhythms, which have been associated with poorer mental health outcomes and suicidal ideation [70,74-76].

#### Eating Disorder

High-risk individuals reported lower appetite and energy, poorer self-care, and more negative thinking (Multimedia Appendix 11), consistent with links between cognitive-emotional dysregulation, somatic symptoms, and disordered eating in adolescents [77,78]. Passive data (Multimedia Appendix 11) showed consistently elevated screen brightness and earlier app use in the high-risk group, possibly indicating compulsive nighttime device use and disrupted sleep-wake cycles, both associated with emotional dysregulation and body image concerns [79,80].

### Strengths and Limitations

This study offers several strengths that advance the field of adolescent digital mental health. By integrating active self-reports and passive smartphone sensor data via the Mindcraft app, we provide a scalable, unobtrusive, and practical framework for early risk detection. Notably, our models maintained strong performance despite high attrition in active data, underscoring the robustness and low participant burden of passive sensing. Additionally, our use of contrastive learning to stabilize day-to-day behavioral features enhanced model robustness. SHAP-based interpretability increased transparency and clinical relevance, both of which are key attributes for adoption in real-world settings.

Several limitations warrant consideration. First, the sample was relatively small and drawn from 3 London-based schools, which may limit the generalizability of our findings, particularly regarding socioeconomic and regional representativeness. Prior research has shown that digital phenotyping features, such as smartphone usage patterns and affective expression, as well as the manifestation and reporting of mental health symptoms, can differ by gender, geography, and cultural context [81-83]. Our geographic concentration in an urban, high-resource setting may therefore not capture behavioral or contextual variability observed in rural or culturally distinct environments, where access to technology, school structures, and sociocultural norms may differ substantially. Furthermore, the gender imbalance driven by the inclusion of a girls-only school may have biased the learned representations toward behavioral and emotional patterns more characteristic of female adolescents, potentially reducing performance for male or nonbinary young people. Consequently, while our findings provide novel insights into adolescents’ behavioral monitoring, caution is warranted when extrapolating these results to nonurban, gender-balanced, and resource-limited populations. Future work should therefore include socioeconomically and demographically diverse samples to assess the generalizability of this approach more robustly.

Second, passive sensor data quality varied across device types, operating systems, permission settings, and user engagement, with some participants not enabling key sensors, such as location or app usage, resulting in heterogeneous data completeness. We applied sentinel values to flag missing sensor inputs, enabling the model to learn from patterns of missingness. However, this may not fully eliminate systematic differences, as participants with more complete data may differ meaningfully from those with limited data, potentially skewing model learning. Future work should incorporate fairness-aware modeling and stratified evaluation to ensure equitable performance across subgroups [84]. While cumulative median aggregation improves robustness to short-term noise, it may dampen sensitivity to clinically meaningful abrupt behavioral changes, such as those observed during acute mood episodes or crisis events. Hybrid strategies such as combining cumulative medians with volatility-sensitive features may better capture both stable patterns and sudden shifts.

Finally, this study did not include broader biological and environmental factors, such as genetic risk, socioeconomic status, family history, adverse childhood experiences, or neurodevelopmental profiles, that are known to influence adolescent mental health [81,85]. The absence of such contextual and historical information may limit the model’s ability to capture all relevant sources of variance, potentially constraining predictive accuracy. Future studies should consider incorporating these factors to enhance explanatory power and clinical utility.

### Implications and Recommendations

Active data engagement declined markedly by day 14 (Figure 2B), underscoring a key longitudinal feasibility challenge. Future iterations of Mindcraft will therefore incorporate specific design adaptations to reduce burden and sustain engagement. These include shifting from fixed-time prompts to context-aware adaptive sampling triggered by behavioral anomalies detected through passive data; incorporating light gamification (such as streaks) to maintain motivation; and closing the feedback loop by providing personalized behavioral insights and just-in-time recommendations informed by each user’s active and passive data. Together, these adaptations aim to shift Mindcraft from a one-way data collection tool to a personalized support platform, thereby improving long-term engagement and feasibility.

Real-time digital phenotyping at a population level can complement traditional screening methods by identifying and prioritizing high-risk individuals and enabling tailored prevention and early intervention strategies [76,86]. The use of a mobile app for digital phenotyping is particularly valuable for children and young people, for whom early identification and intervention are essential to prevent the onset of more severe mental health issues in adulthood. When implemented in schools, it addresses barriers such as stigma and accessibility, offering adolescents a preventive tool that empowers them to manage their mental health. Digital phenotyping provides the opportunity to inform school-based digital interventions that might be central to early intervention and prevention of mental health problems in the community [87]. While adherence to GDPR and secure data protocols provides a legal baseline, the ethical landscape of adolescent digital phenotyping extends far beyond regulatory compliance [88,89]. Collecting continuous passive data from minors introduces distinct tensions regarding autonomy and informed consent; specifically, the invisible nature of passive sensing means adolescents may habituate to the monitoring, potentially eroding their ongoing awareness of data sharing [89]. Furthermore, the deployment of predictive risk models carries the risk of digital labeling, where algorithmic outputs, if misinterpreted or generating false positives, could lead to stigma, unnecessary anxiety, or oversurveillance [88]. Addressing these complexities demands more than static consent forms; it requires ongoing participatory approaches involving adolescents, parents, and clinicians, alongside governance mechanisms that ensure transparency, prioritize interpretability over black-box predictions, and maintain human clinical oversight of algorithmic outputs [88]. To this end, future work must empirically evaluate how adolescents understand, experience, and respond to continuous passive sensing, and determine how ethical frameworks can best support safe, acceptable integration in school settings.

Achieving real-world feasibility requires distinguishing between scientific interpretability and user-centric explainability [90,91]. While the SHAP values presented in this study provide necessary transparency for model validation, raw feature importance scores are unlikely to be meaningful to non-expert stakeholders such as adolescents, parents, or educators. For broad deployment, these technical outputs must be bridged by a translation layer that converts granular risk estimates into accessible, actionable narratives. For instance, rather than displaying a high SHAP value for “location entropy,” a user-facing interface should translate this into an intuitive insight, such as detecting “significant changes in your daily routine.” Future implementation work must prioritize the co-design of these explanatory interfaces to ensure that algorithmic transparency supports understanding rather than overwhelming users.

Digital biomarkers from sensors and ML have shown accuracy in predicting disease progression [92,93], underscoring the broader potential of sensor-based technologies for personalized healthcare. Smartphones, being both ubiquitous and affordable, enable continuous, real-time data collection even in low-resource settings, reducing reliance on clinical supervision [34,94]. Digital phenotyping offers a scalable mechanism for continuous, context-aware monitoring [94], which could feed into well-being dashboards accessible to pastoral staff, school counselors, or clinical teams. In school settings, such dashboards could support early identification of distress and enable stepped-care approaches that match support intensity to need. In clinical pathways, risk predictions could support triage decisions and monitoring between appointments, complementing existing services rather than replacing them [86]. Achieving this will require implementation research on workflow integration, governance, and alignment with school well-being policies and child mental health services.

Traditional platforms such as Childline rely on proactive engagement from children and young people, creating barriers for disengaged users. In contrast, Mindcraft’s passive tracking capabilities offer a proactive approach by identifying early signs of poor mental health and prompting timely professional interventions. Building on this pilot work, we have developed a school-based intervention study that evaluates the effectiveness of personalized artificial intelligence recommendations delivered through the Mindcraft app across schools in the United Kingdom (ISRCTN11686798). With this further development, Mindcraft is evolving into a comprehensive platform that delivers in-app recommendations informed by active and passive data, leveraging user profiles [95,96] to tailor suggestions to individual needs, enhance engagement, and improve intervention effectiveness. Subsequent phases will focus on scaling and validation across diverse school settings and on integrating Mindcraft with existing education and mental health pathways to support sustainable, real-world implementation. This integration of proactive detection and tailored intervention could help address significant gaps in traditional mental health support systems.

### Conclusion

In conclusion, this study underscores the transformative potential of integrating active and passive smartphone data to predict adolescent mental health. By leveraging innovative ML techniques, such as contrastive learning, and the scalability of tools like the Mindcraft app, we present a robust framework for early risk detection across diverse mental health outcomes. These findings lay the groundwork for more inclusive, accessible, and personalized early detection and intervention strategies in adolescent mental health.

## Supplementary material

10.2196/72501 Multimedia Appendix 1 Active data features, including feature descriptions and Spearman correlations with mental health outcomes (SDQ, SCI, ED-15, and suicidal ideation).

10.2196/72501 Multimedia Appendix 2 Passive data features engineered from smartphone sensors, including feature descriptions and Spearman correlations with mental health outcomes (SDQ, SCI, ED-15, and suicidal ideation).

10.2196/72501 Multimedia Appendix 3 Example illustrating the behavior of the cumulative median compared to raw daily step count values for a single participant. The cumulative median smooths isolated anomalies (eg, a one-day spike on Day 3) while remaining sensitive to sustained behavioral shifts (eg, a persistent drop beginning on Day 7).

10.2196/72501 Multimedia Appendix 4 Balanced accuracy (mean [SD] across 10 different runs of the experiment) for models trained on raw daily features, cumulative mean-aggregated and cumulative median–aggregated features of the combined active and passive data for the mental health outcomes.

10.2196/72501 Multimedia Appendix 5 CONSORT (Consolidated Standards of Reporting Trials)-style flow diagram showing participant inclusion for the main analysis. The main analysis included 67 users who provided at least one active self-report and enabled at least one passive data stream.

10.2196/72501 Multimedia Appendix 6 Distribution of mental health assessment scores across participants. (A) Strengths and Difficulties Questionnaire (SDQ) scores, representing overall mental health and behavioral difficulties. (B) Sleep Condition Indicator (SCI) scores, assessing sleep quality and potential insomnia. (C) Frequency of self-reported suicidal ideations over a 2-week period. (D) Eating Disorder Examination Questionnaire (ED-15) scores, evaluating symptoms associated with eating disorders.

10.2196/72501 Multimedia Appendix 7 Fully annotated correlation heatmap of active data features, with Spearman correlation coefficients displayed in each cell. This supplementary version is provided to support the complete numerical transparency of the simplified heatmap shown in Figure 3B of the main manuscript.

10.2196/72501 Multimedia Appendix 8 Mean (SE) *z* score–normalized values of the top five active and passive features that significantly differed between low- and high-risk groups across 4 mental health outcomes: SDQ, insomnia, suicidal ideation, and eating disorder. Group differences were assessed using one-sided t-tests with Benjamini–Hochberg correction for multiple comparisons. Features were selected based on adjusted p-values. Red asterisks indicate statistical significance (*P≤.05, **P≤.01, ***P≤.001). Normalization was applied to enable comparison across features measured on different scales.

10.2196/72501 Multimedia Appendix 9 Comparison of balanced accuracy of mental health outcome predictions using active data only for all users in the study (N=103) vs users who also enabled passive data collection (N=67).

10.2196/72501 Multimedia Appendix 10 Detailed performance metrics for predicting SDQ high risk, insomnia, suicidal ideation, and eating disorder risk using combined active and passive data in an external validation sample (N=45 adolescents not used for model development).

10.2196/72501 Multimedia Appendix 11 Fairness analysis across gender and school context for the combined active and passive data model. Balanced accuracy for predicting SDQ high risk, insomnia, suicidal ideation, and eating disorder risk stratified by (A) gender (female vs male/other) and (B–D) school context. Error bars represent SD across 10 different runs of the experiment.

10.2196/72501 Multimedia Appendix 12 Fairness analysis of the combined active and passive data model across passive-sensor availability. Balanced accuracy for users with less than or equal to 3 enabled sensors and greater than 3 enabled sensors for each mental health outcome. Error bars represent the SD across 10 different runs of the experiment.

10.2196/72501 Multimedia Appendix 13 Feature importance analysis for predicting the insomnia high-risk category using both active and passive data. (A) SHAP-based feature importances, with passive data aggregated by sensor type and active data shown individually. (B) Distribution of the top five active data features across insomnia risk categories. (C) Distribution of the top five passive data features across insomnia risk categories. Statistically significant differences between low-risk and high-risk groups are indicated (*P<.05, **P<.01, ***P<.001, *t* test).

10.2196/72501 Multimedia Appendix 14 Feature importance analysis for predicting the suicidal ideation high-risk category using both active and passive data. (A) SHAP-based feature importances, with passive data aggregated by sensor type and active data shown individually. (B) Distribution of the top five active data features across suicidal ideation risk categories. (C) Distribution of the top five passive data features across suicidal ideation risk categories. Statistically significant differences between low-risk and high-risk groups are indicated (*P<.05, **P<.01, ***P<.001, *t* test).

10.2196/72501 Multimedia Appendix 15 Feature importance analysis for predicting the eating disorder high-risk category using both active and passive data. (A) SHAP-based feature importances, with passive data aggregated by sensor type and active data shown individually. (B) Distribution of the top five active data features across eating disorder risk categories. (C) Distribution of the top five passive data features across eating disorder risk categories. Statistically significant differences between low-risk and high-risk groups are indicated (*P<.05, **P<.01, ***P<.001, *t* test).

## Abbreviations

AUC: area under the receiver operating characteristic curve
CONSORT: Consolidated Standards of Reporting Trials
ED-15: Eating Disorder-15 questionnaire
GDPR: General Data Protection Regulation
LOSO CV: leave-one-subject-out cross-validation
ML: machine learning
MLP: multilayer perceptron
PHQ-9: Patient Health Questionnaire version 9
SCI: Sleep Condition Indicator
SDQ: Strengths and Difficulties Questionnaire
SES: socioeconomic status
SHAP: Shapley Additive Explanations
