# Supplementary material for: Digital Phenotyping for Adolescent Mental Health: Feasibility Study Using Machine Learning to Predict Mental Health Risk From Active and Passive Smartphone Data
Source: J Med Internet Res. 2026 Feb 4;28:e72501. doi: 10.2196/72501 (PMC12871944; doi:10.2196/72501)
Supplement: Multimedia Appendix 1 [file jmir-v28-e72501-s001.docx]

**Supplementary Table 1:** Active data features, including feature descriptions and Spearman correlations with mental health outcomes (SDQ, SCI, ED-15, and suicidal ideation). Statistical significance for Spearman correlations is denoted as follows: * : p < 0.05, ** : p < 0.01, and *** : p < 0.001.

| **Feature** | **SDQ** | **SCI** | **Suicidal ideation** | **ED-15** |
| --- | --- | --- | --- | --- |
| Appetite - How is your appetite today? | -0.32*** | 0.40*** | -0.24*** | -0.36*** |
| Confidence - How confident do you feel today? | -0.44*** | 0.30*** | -0.34*** | -0.42*** |
| Energy Levels - How are your energy levels today? | -0.33*** | 0.22*** | -0.26*** | -0.31*** |
| Exercise - How many hours and minutes have you exercised today? | -0.24*** | 0.14** | -0.22*** | -0.09 |
| Headaches - How badly have you suffered a headache today? | 0.11* | -0.20*** | 0.09 | 0.18*** |
| Hopefulness - How hopeful do you feel today? | -0.43*** | 0.33*** | -0.35*** | -0.34*** |
| Irritability - Do you feel irritable today? | 0.35*** | -0.40*** | 0.37*** | 0.40*** |
| Leisure - Are you able to engage in an activity you enjoy today? | -0.26*** | 0.35*** | -0.16** | -0.29*** |
| Loneliness - How lonely are you feeling today? | 0.48*** | -0.42*** | 0.46*** | 0.31*** |
| Mood - How are you feeling today? | -0.35*** | 0.30*** | -0.39*** | -0.35*** |
| Motivation - How is your motivation today? | -0.27*** | 0.20*** | -0.25*** | -0.19*** |
| Negative thinking - How negative do you think today? | 0.48*** | -0.47*** | 0.57*** | 0.46*** |
| Productivity - Are you feeling productive today? | -0.23*** | 0.22*** | -0.12* | -0.20*** |
| Racing Thoughts - Are you experiencing racing thoughts today? | 0.45*** | -0.44*** | 0.52*** | 0.48*** |
| Self Care - How is your self-care today? | -0.37*** | 0.33*** | -0.24*** | -0.42*** |
| Sleep quality - How did you sleep last night? | -0.37*** | 0.44*** | -0.34*** | -0.20*** |
| Sleep quantity - How many hours and minutes of sleep did you get last night? | -0.12* | 0.18*** | 0 | 0.01 |
| Sociability - How sociable do you feel today? | -0.38*** | 0.33*** | -0.29*** | -0.28*** |
| Your Measure - Do you have something you want to track today? | -0.30*** | 0.17** | -0.09 | -0.11 |
