# Supplementary material for: Digital Phenotyping for Adolescent Mental Health: Feasibility Study Using Machine Learning to Predict Mental Health Risk From Active and Passive Smartphone Data
Source: J Med Internet Res. 2026 Feb 4;28:e72501. doi: 10.2196/72501 (PMC12871944; doi:10.2196/72501)
Supplement: Multimedia Appendix 2 [file jmir-v28-e72501-s002.docx]

**Supplementary Table 2:** Passive data features engineered from smartphone sensors, including feature descriptions and Spearman correlations with mental health outcomes (SDQ, SCI, ED-15, and suicidal ideation). For the purposes of feature extraction, night-time was defined as the interval from 22:00 to 06:00. Statistical significance for Spearman correlations is denoted as follows: * : p < 0.05, ** : p < 0.01, and *** : p < 0.001.

| **Feature** | **SDQ** | **SCI** | **Suicidal ideation** | **ED-15** |
| --- | --- | --- | --- | --- |
| Total number of steps taken during the full day | 0.01 | -0.04 | 0.02 | 0 |
| Whether more than 5,000 steps were taken | 0.01 | -0.06 | 0.04 | 0.01 |
| Whether more than 7500 steps were taken | 0.08 | -0.11 | 0.05 | 0.05 |
| Whether more than 1000 steps were taken | 0.06 | -0.04 | 0 | -0.06 |
| Mean latitude of GPS samples over the full day | 0.38* | -0.23 | 0.44** | -0.03 |
| Mean longitude of GPS samples over the full day | 0.19 | -0.38* | 0.25 | -0.01 |
| Sum of distance travelled over the full day | 0.29 | -0.22 | 0.25 | 0.05 |
| Number of locations visited over the full day | 0.31 | -0.25 | 0.23 | 0.08 |
| Farthest GPS point from the daily centroid | 0.19 | -0.12 | 0.16 | -0.08 |
| Mean distance of GPS points from the centroid | 0.14 | -0.08 | 0.13 | -0.11 |
| Median distance of GPS points from the centroid | 0.14 | -0.08 | 0.13 | -0.09 |
| Nighttime movement | 0.16 | -0.14 | 0.16 | -0.06 |
| Spatial dispersion (A measure of how far, on average, a person moves from their typical location over the full day) | 0.15 | -0.09 | 0.14 | -0.1 |
| Standard deviation of latitude values | 0.19 | -0.12 | 0.16 | -0.06 |
| Standard deviation of longitude values | 0.12 | -0.07 | 0.11 | -0.11 |
| Shannon entropy of visited GPS locations (A measure of how varied and unpredictable a person’s locations were over the full day) | 0.24 | -0.17 | 0.13 | 0.06 |
| Minutes spent at the inferred home location | 0.16 | -0.17 | 0.12 | -0.33 |
| Count of distinct apps opened | 0.16 | -0.16 | -0.03 | 0.2 |
| Total number of app-foreground events | 0.19 | -0.12 | 0.18 | 0.28 |
| Total time spent using apps during the day | 0.27 | -0.14 | 0.41** | 0.24 |
| Mean app session duration over the full day | 0.21 | -0.06 | 0.38** | 0.16 |
| Median app session duration over the full day | 0.37* | -0.19 | 0.44*** | 0.19 |
| Number of camera app usage events over the full day | 0.04 | -0.05 | -0.14 | -0.03 |
| Number of communication app usage events over the full day | 0.02 | 0 | 0.22 | -0.05 |
| Number of entertainment app usage events over the full day | 0.37** | -0.37* | 0.51*** | 0.41** |
| Number of gaming app usage events over the full day | -0.22 | -0.02 | -0.09 | -0.13 |
| Number of mental health app usage events over the full day | 0.33 | -0.34* | -0.01 | 0.26 |
| Number of Mindcraft app was used | -0.13 | 0.24 | -0.12 | 0.1 |
| Number of news app usage events over the full day | 0.07 | 0.08 | 0.17 | -0.09 |
| Number of other app usage events over the full day | 0.01 | 0.1 | -0.05 | 0.04 |
| Number of physical health app usage events over the full day | -0.04 | 0 | -0.22 | 0.16 |
| Number of productivity app usage events over the full day | 0.33 | -0.17 | 0.26 | 0.21 |
| Number of social media app usage events over the full day | 0.19 | -0.18 | 0.1 | 0.26 |
| Percent of app usage time spent on the camera apps | -0.03 | -0.02 | -0.14 | -0.1 |
| Percent of app usage time spent on the communication apps | -0.01 | 0.04 | 0.18 | -0.08 |
| Percent of app usage time spent on the entertainment apps | 0.27 | -0.24 | 0.37** | 0.24 |
| Percent of app usage time spent on the gaming apps | -0.2 | -0.06 | -0.11 | -0.17 |
| Percent of app usage time spent on the mental health apps | 0.32 | -0.35* | -0.05 | 0.25 |
| Percent of app usage time spent on the Mindcraft app | -0.22 | 0.28 | -0.17 | -0.06 |
| Percent of app usage time spent on the news apps | 0.07 | 0.09 | 0.17 | -0.09 |
| Percent of app usage time spent on the other apps | -0.14 | 0.29 | -0.38** | -0.35* |
| Percent of app usage time spent on the physical health apps | -0.03 | -0.01 | -0.22 | 0.16 |
| Percent of app usage time spent on the productivity apps | 0.33 | -0.2 | 0.22 | 0.18 |
| Percent of app usage time spent on the social media apps | -0.03 | 0.03 | -0.22 | 0.05 |
| Percent of app usage time spent on the mental health apps | 0.17 | -0.21 | -0.16 | 0.28 |
| Percent of app usage time spent on the mental and physical health apps | 0.16 | -0.22 | -0.21 | 0.28 |
| Number of different apps used at night | 0.1 | -0.1 | 0.07 | 0.31 |
| Total Number of app usage events at night | 0.09 | -0.06 | 0.27 | 0.39** |
| Total time spent using apps at night | 0.15 | -0.09 | 0.37** | 0.28 |
| Mean time an app was used per event at night | 0.07 | -0.02 | 0.31 | 0 |
| Median time an app was used per event at night | 0.19 | -0.12 | 0.42*** | 0.07 |
| Total background noise over the full day | -0.13 | 0.21 | 0.02 | -0.06 |
| Median background noise levels over the full day | -0.2 | 0.05 | 0.01 | 0.18 |
| Mean background noise levels over the full day | 0 | -0.08 | 0.25 | 0.34 |
| Max background noise levels over the full day | -0.49** | 0.36 | -0.39 | -0.1 |
| Standard deviation of background noise levels over the full day | -0.43 | 0.36 | -0.63*** | -0.31 |
| Total background noise levels at night | 0.11 | -0.01 | 0.29 | 0.12 |
| Median background noise levels at night | 0.23 | -0.19 | 0.43 | 0.39 |
| Mean background noise levels at night | 0.2 | -0.23 | 0.42 | 0.46* |
| Max background noise levels at night | 0.04 | -0.09 | 0.02 | 0.15 |
| Standard deviation of background noise levels at night | -0.22 | 0.14 | -0.38 | -0.13 |
| Minimum battery level (%) over the full day | -0.09 | 0.11 | 0.02 | 0.06 |
| Maximum battery level (%) over the full day | -0.12 | 0.16 | -0.09 | 0.03 |
| Mean battery level (%) over the full day | -0.15 | 0.17* | -0.05 | 0.06 |
| Median battery level (%) over the full day | -0.17 | 0.17 | -0.06 | 0.06 |
| Number of charging episodes over the full day | 0.07 | 0.03 | 0.03 | 0.02 |
| Mean battery discharge rate (% per hour) | 0.03 | -0.04 | -0.01 | -0.02 |
| Duration with battery < 20% | 0.13 | -0.15 | -0.02 | -0.01 |
| Number of battery data samples collected at night | -0.02 | 0.04 | -0.16 | 0.02 |
| Earliest hour the Mindcraft was opened in a day | -0.11 | 0.12 | -0.16 | -0.18 |
| Latest hour the Mindcraft app was opened in a day | -0.14 | 0.19 | -0.14 | -0.1 |
| Indicator that the Mindcraft app was opened at night | -0.08 | 0 | -0.05 | 0.11 |
| Total screen brightness levels over the full day | 0.12 | 0.07 | 0.02 | -0.03 |
| Median screen brightness levels over the full day | -0.01 | 0.02 | -0.06 | 0.06 |
| Mean screen brightness levels over the full day | 0.05 | 0.04 | -0.03 | 0.04 |
| Standard deviation of screen brightness levels over the full day | 0.16 | 0.16 | 0.08 | -0.12 |
| Total screen brightness levels at night | 0.12 | -0.11 | 0.08 | -0.21 |
| Median screen brightness levels at night | 0.08 | -0.03 | 0.02 | -0.17 |
| Mean screen brightness levels at night | 0.11 | -0.08 | 0.06 | -0.21 |
| Standard deviation of screen brightness levels at night | 0.16 | -0.12 | 0.18 | -0.22 |
| Total ambient light over the full day | 0.24 | -0.2 | 0.2 | 0.23 |
| Median ambient light over the full day | 0.12 | -0.33 | 0.25 | 0.2 |
| Mean ambient light over the full day | 0.31 | -0.34* | 0.27 | 0.31 |
| Standard deviation of ambient-light levels | 0.32 | -0.33 | 0.24 | 0.3 |
| Total ambient light at night | 0.09 | -0.19 | 0.13 | 0.29 |
| Median ambient light at night | 0.06 | -0.39** | 0.14 | 0.27 |
| Mean ambient light at night | 0.13 | -0.35* | 0.19 | 0.36* |
| Standard deviation of ambient-light levels at night | 0.19 | -0.3 | 0.17 | 0.27 |
