# Supplementary figures and images for: Digital Phenotyping for Adolescent Mental Health: Feasibility Study Using Machine Learning to Predict Mental Health Risk From Active and Passive Smartphone Data
Source: J Med Internet Res. 2026 Feb 4;28:e72501. doi: 10.2196/72501 (PMC12871944; doi:10.2196/72501)

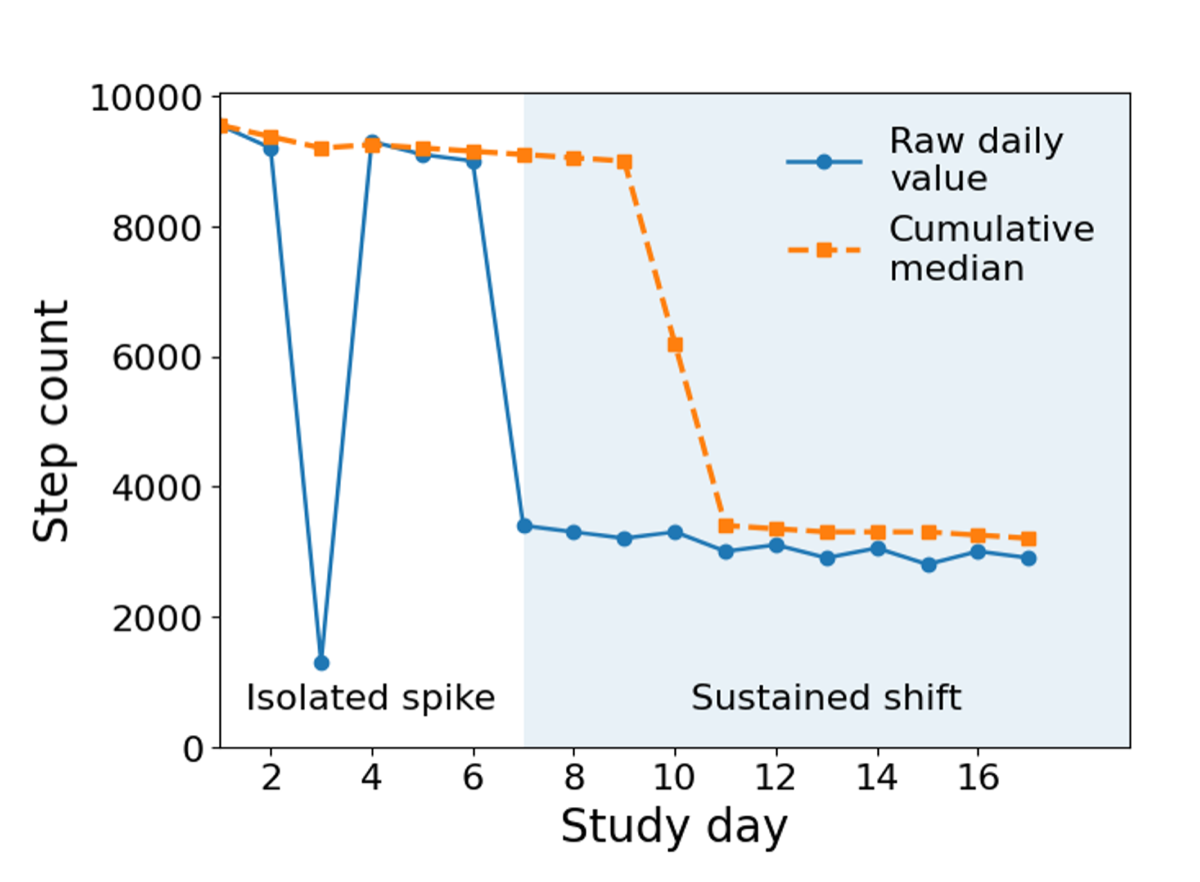

Supplement: Multimedia Appendix 3 [file jmir-v28-e72501-s003.png]

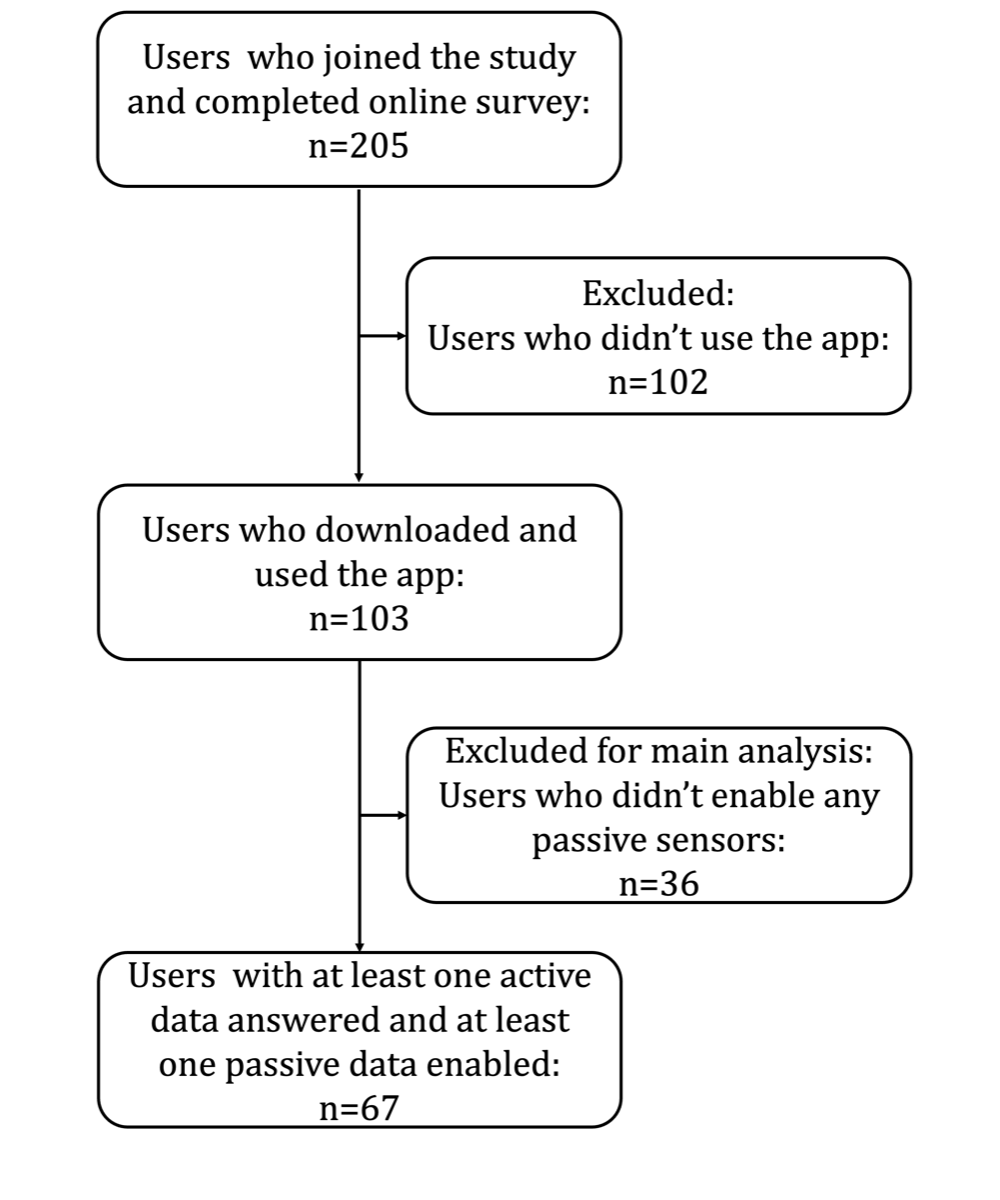

Supplement: Multimedia Appendix 5 [file jmir-v28-e72501-s005.png]

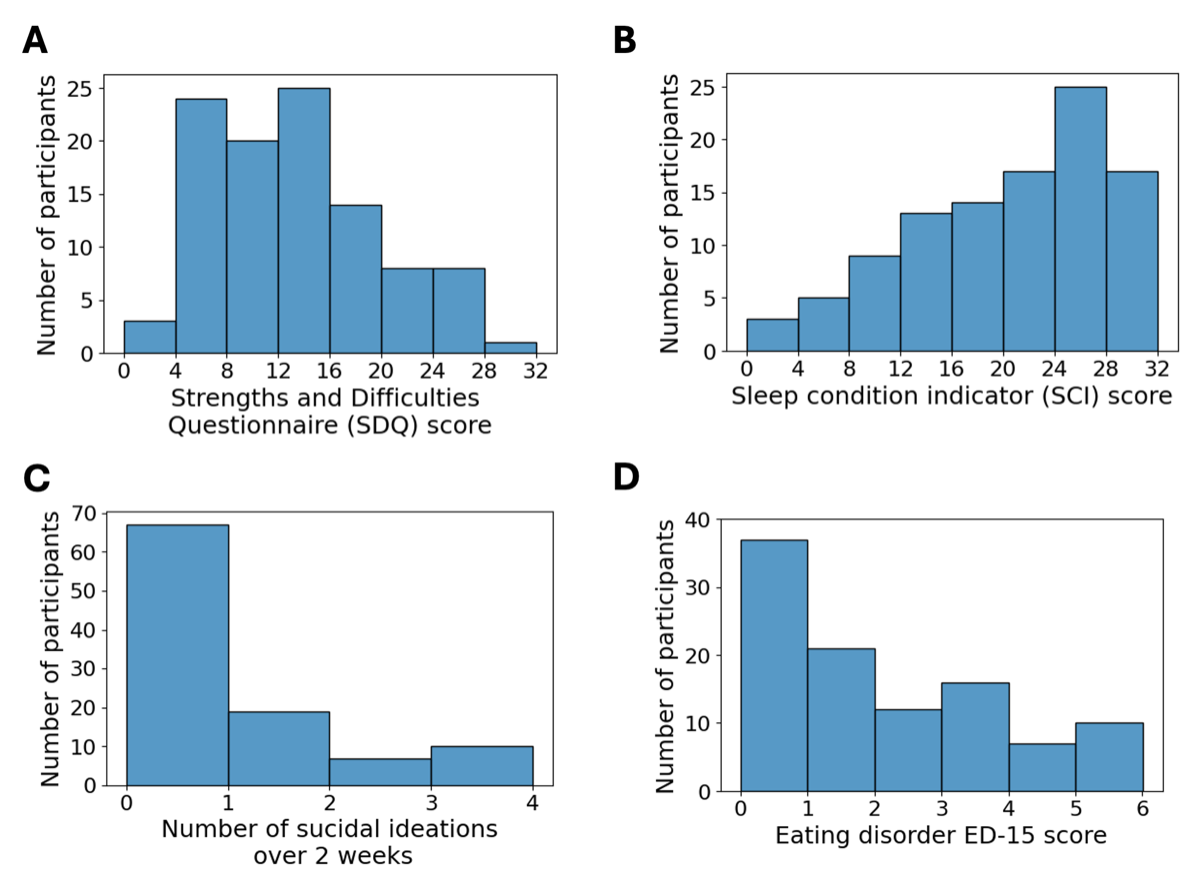

Supplement: Multimedia Appendix 6 [file jmir-v28-e72501-s006.png]

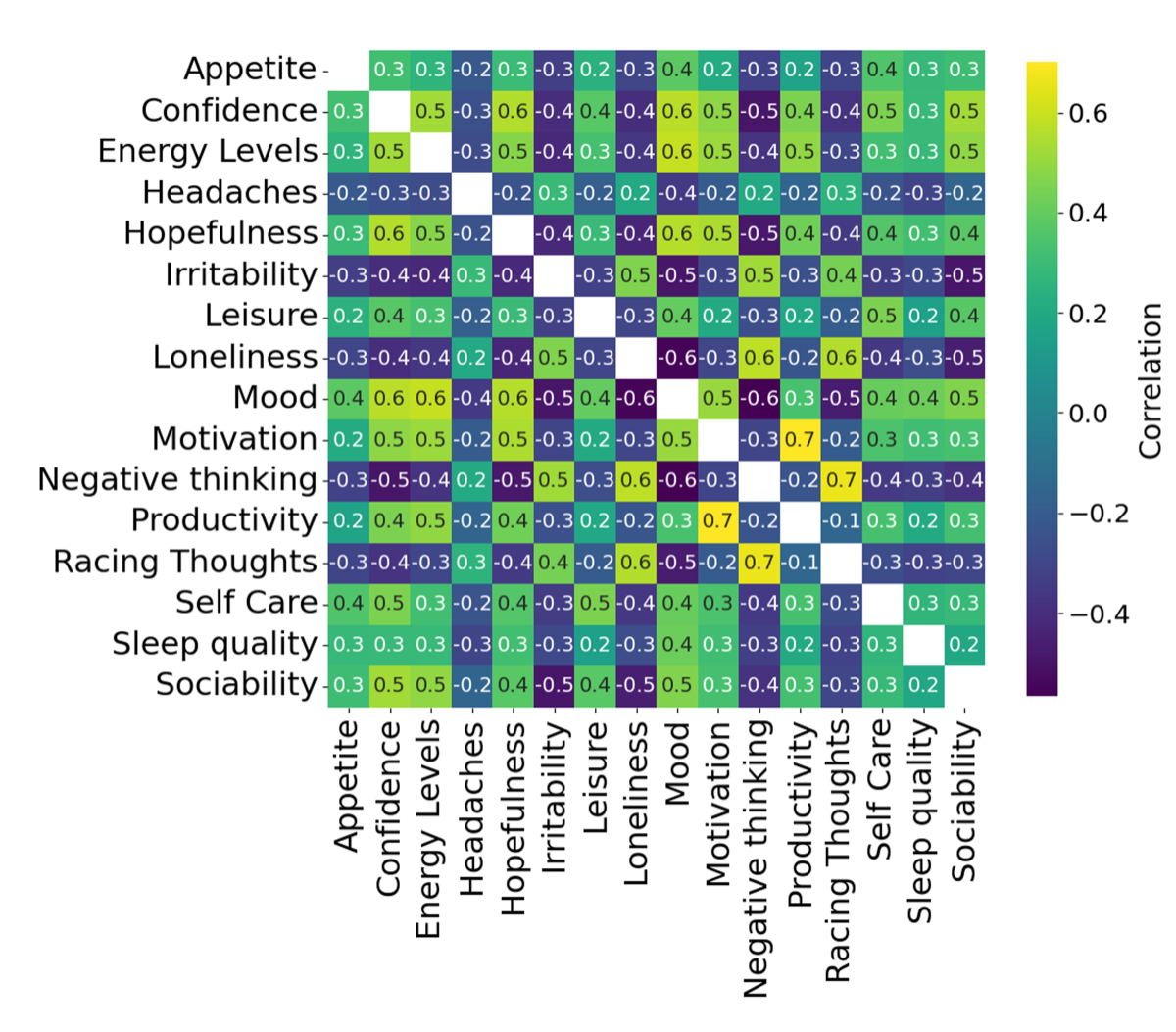

Supplement: Multimedia Appendix 7 [file jmir-v28-e72501-s007.png]

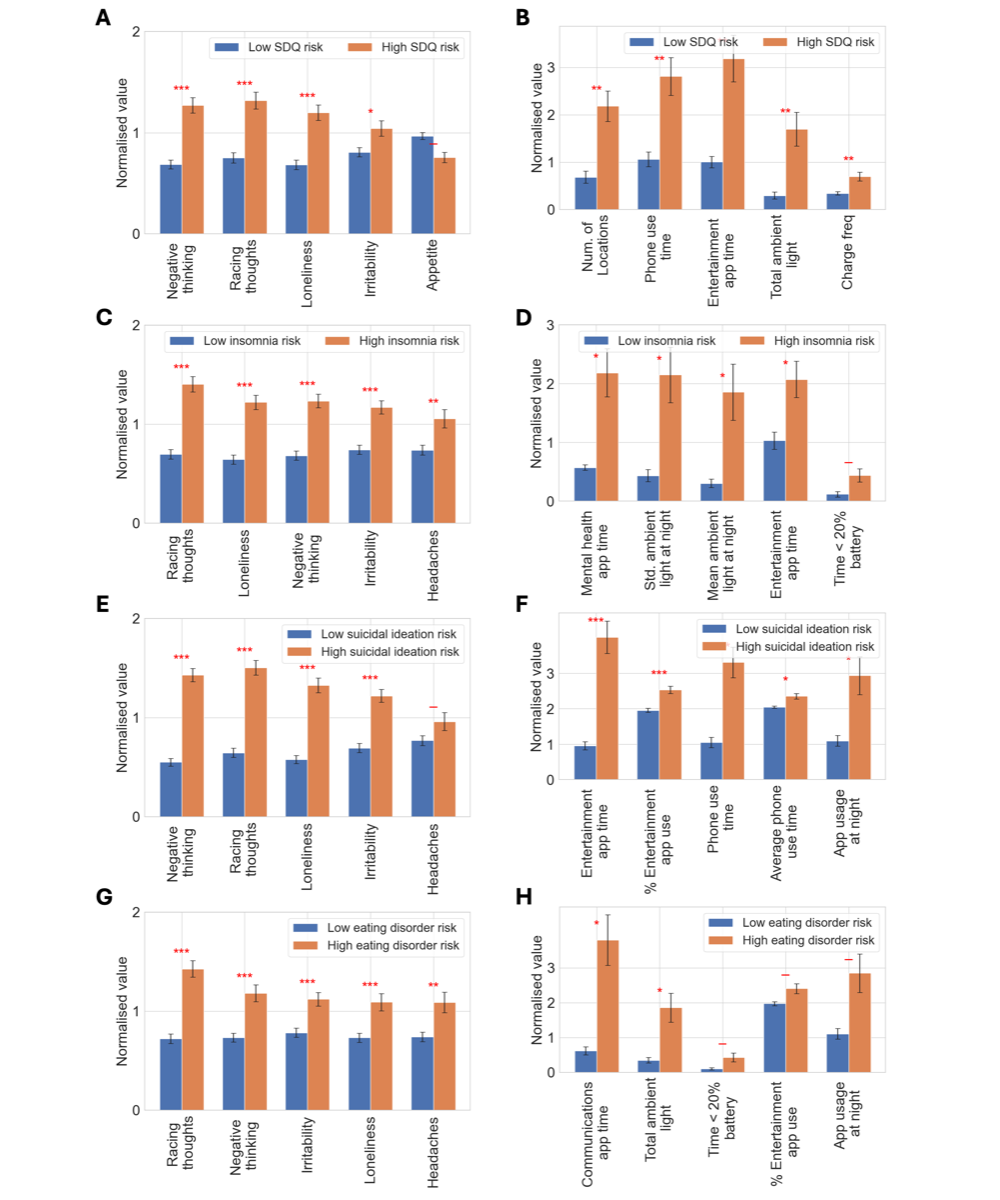

Supplement: Multimedia Appendix 8 [file jmir-v28-e72501-s008.png]

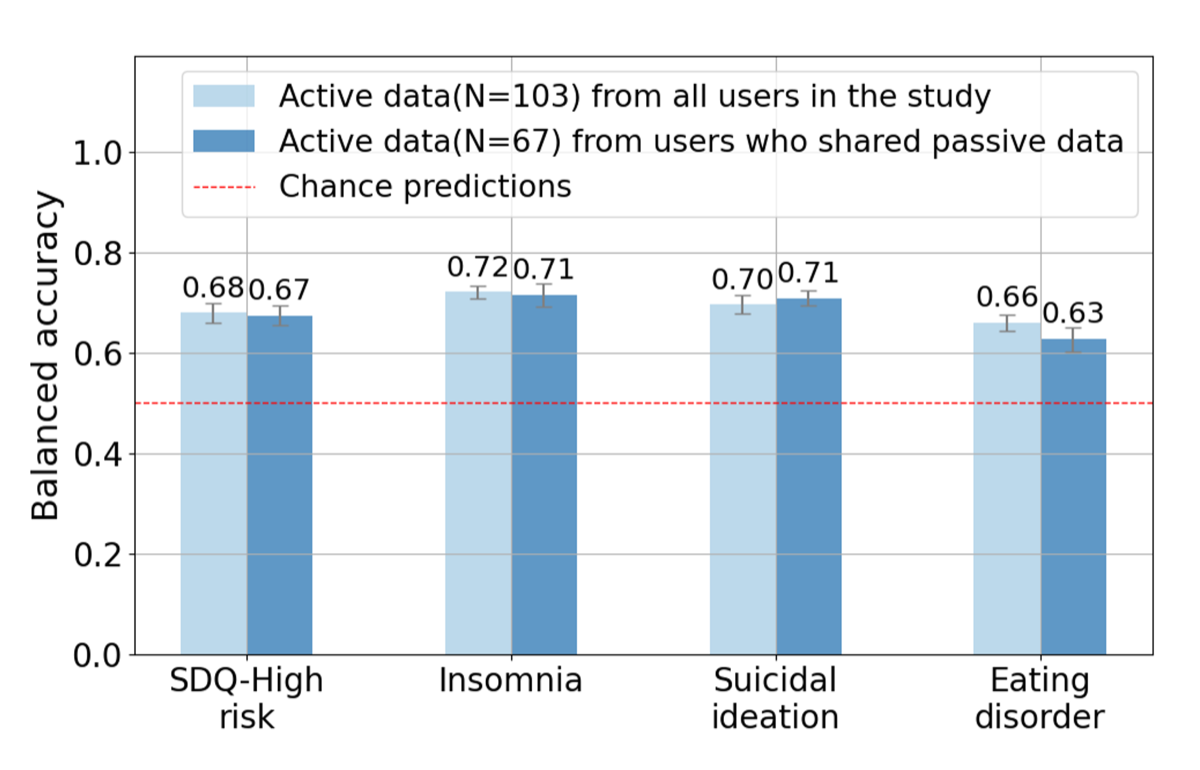

Supplement: Multimedia Appendix 9 [file jmir-v28-e72501-s009.png]

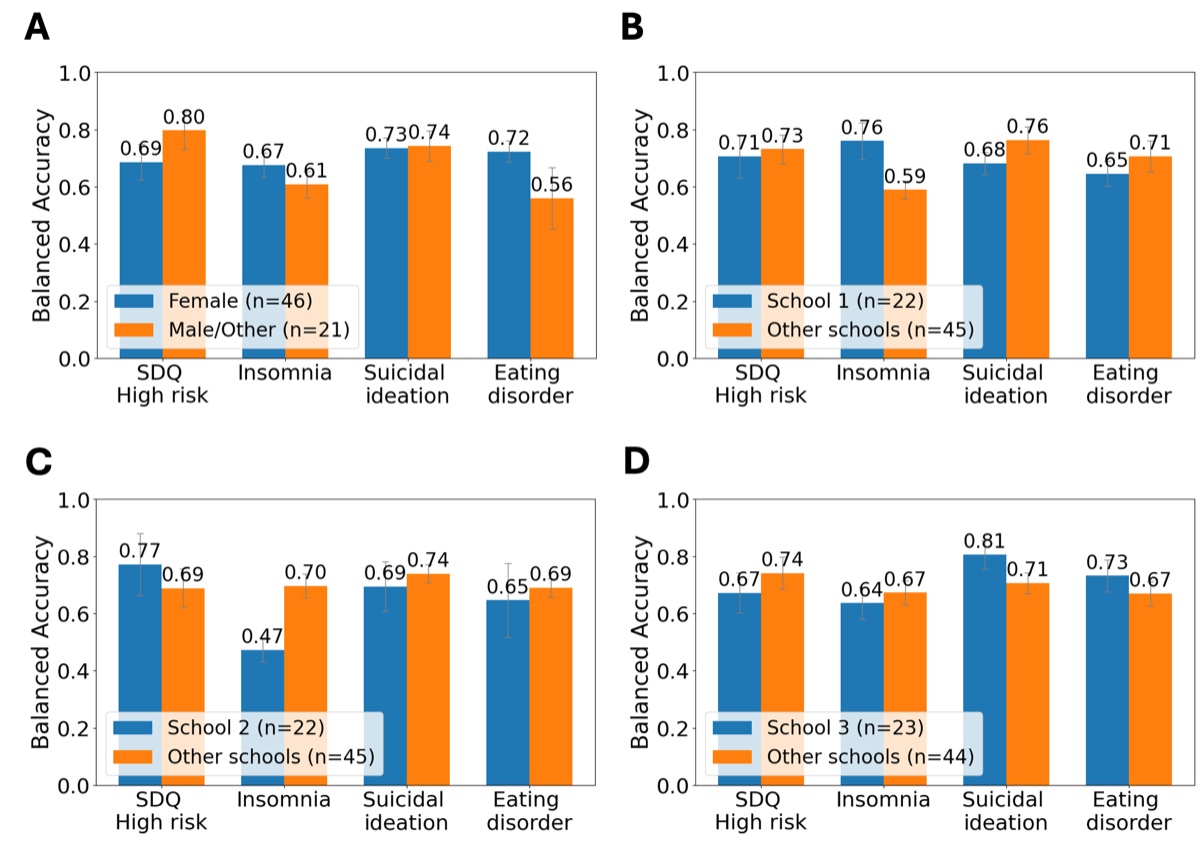

Supplement: Multimedia Appendix 11 [file jmir-v28-e72501-s011.png]

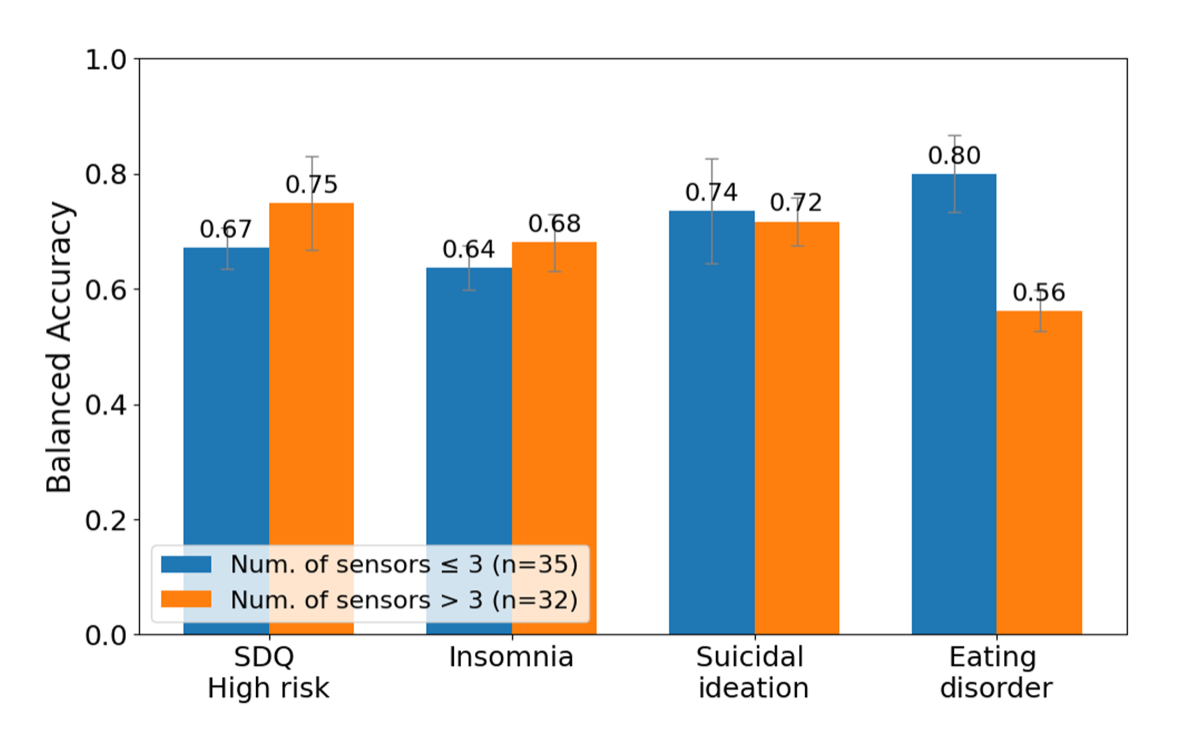

Supplement: Multimedia Appendix 12 [file jmir-v28-e72501-s012.png]

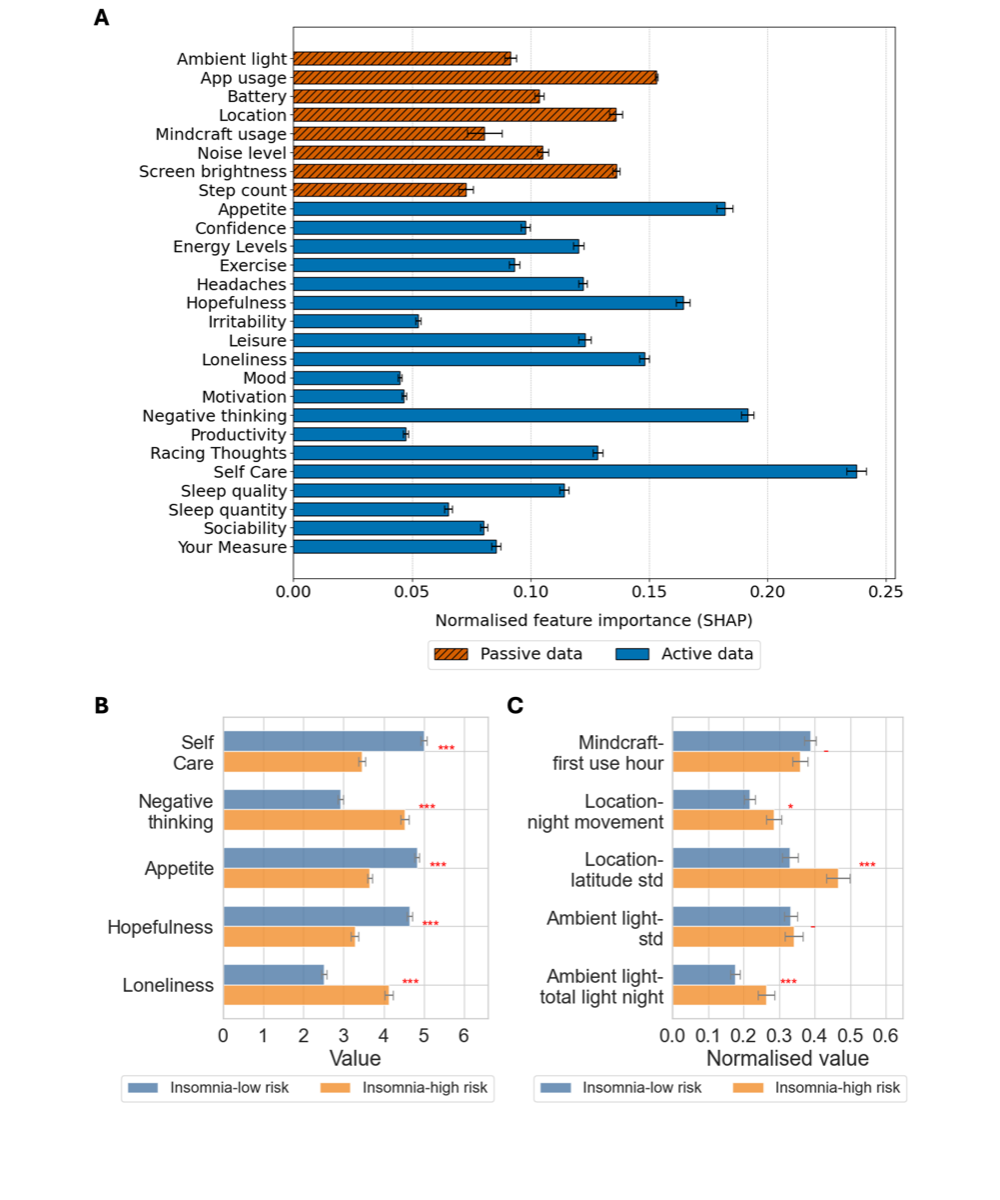

Supplement: Multimedia Appendix 13 [file jmir-v28-e72501-s013.png]

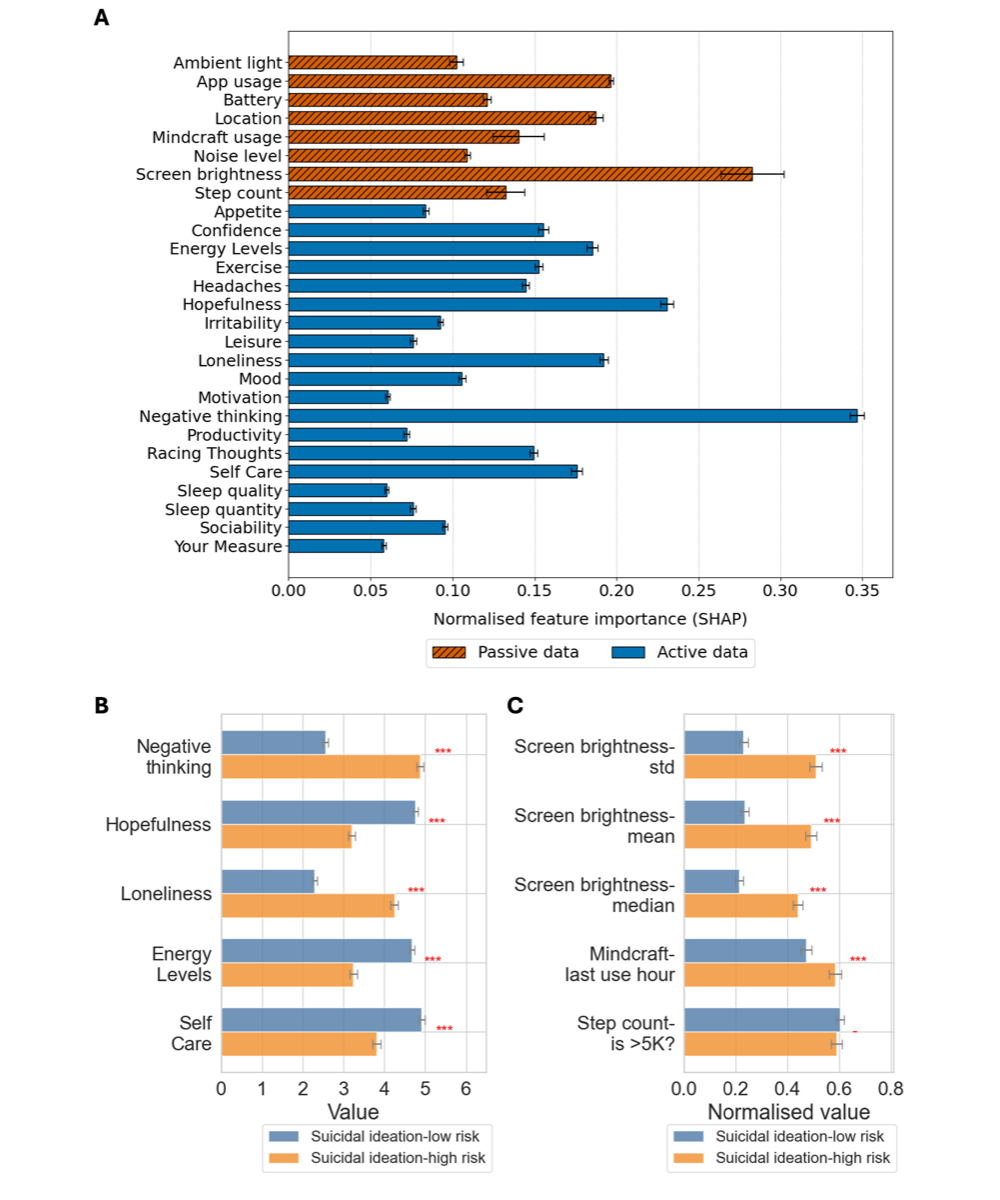

Supplement: Multimedia Appendix 14 [file jmir-v28-e72501-s014.png]

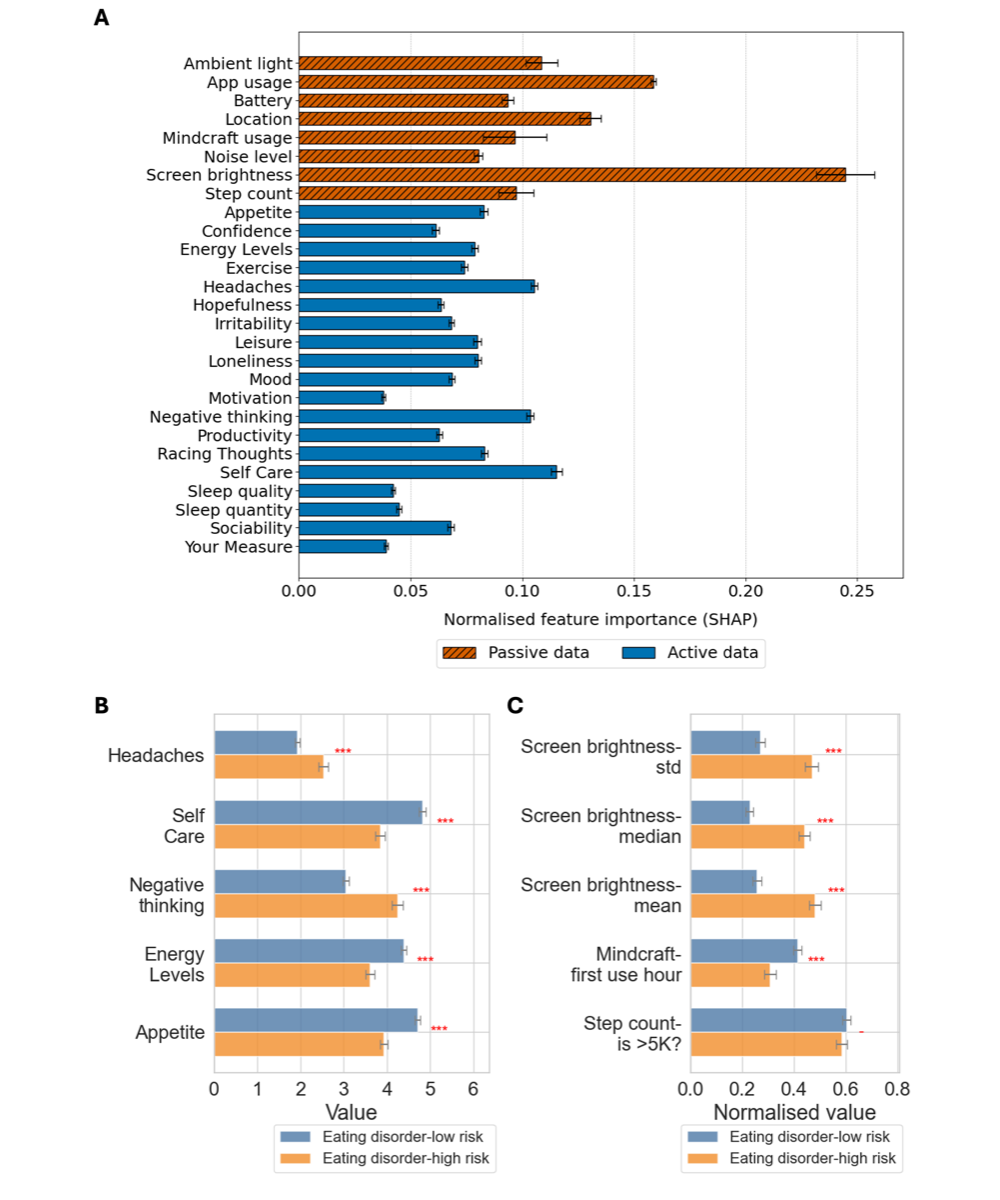

Supplement: Multimedia Appendix 15 [file jmir-v28-e72501-s015.png]
