# Supplementary material for: Digital Phenotyping for Adolescent Mental Health: Feasibility Study Using Machine Learning to Predict Mental Health Risk From Active and Passive Smartphone Data
Source: J Med Internet Res. 2026 Feb 4;28:e72501. doi: 10.2196/72501 (PMC12871944; doi:10.2196/72501)
Supplement: Multimedia Appendix 4 [file jmir-v28-e72501-s004.docx]

**Supplementary Table 3:** Balanced accuracy (mean ± standard deviation across 10 different runs of the experiment) for models trained on raw daily features, cumulative mean-aggregated and cumulative median–aggregated features of the combined active and passive data for the mental health outcomes.

| **Outcome** | **Raw Data** | **Cumulative Mean** | **Cumulative Median** |
| --- | --- | --- | --- |
| SDQ-high risk | 0.51 ± 0.04 | 0.71 ± 0.04 | 0.71 ± 0.03 |
| Insomnia | 0.41 ± 0.03 | 0.66 ± 0.03 | 0.67 ± 0.04 |
| Suicidal ideation | 0.64 ± 0.02 | 0.75 ± 0.04 | 0.77 ± 0.03 |
| Eating disorder | 0.65 ± 0.02 | 0.71 ± 0.05 | 0.70 ± 0.03 |
