# Supplementary material for: Digital Phenotyping for Adolescent Mental Health: Feasibility Study Using Machine Learning to Predict Mental Health Risk From Active and Passive Smartphone Data
Source: J Med Internet Res. 2026 Feb 4;28:e72501. doi: 10.2196/72501 (PMC12871944; doi:10.2196/72501)
Supplement: Multimedia Appendix 10 [file jmir-v28-e72501-s010.docx]

**Supplementary Table 4:** Detailed performance metrics for predicting SDQ high risk, insomnia, suicidal ideation, and eating disorder risk using combined active and passive data in an external validation sample (N = 45 adolescents not used for model development). Results are presented as mean ± standard deviation across 10 repeated runs.

|  | SDQ-High Risk | Insomnia | Suicidal ideation | Eating disorder |
| --- | --- | --- | --- | --- |
| Balanced accuracy | 0.71 ± 0.03 | 0.67 ± 0.04 | 0.77 ± 0.03 | 0.70 ± 0.03 |
| AUC | 0.77 ± 0.03 | 0.74 ± 0.02 | 0.82 ± 0.03 | 0.73 ± 0.02 |
| AUC-PR | 0.53 ± 0.04 | 0.52 ± 0.05 | 0.64 ± 0.05 | 0.52 ± 0.03 |
| F1 | 0.61 ± 0.04 | 0.59 ± 0.04 | 0.70 ± 0.04 | 0.61 ± 0.03 |
| F1 macro | 0.69 ± 0.03 | 0.66 ± 0.04 | 0.76 ± 0.03 | 0.68 ± 0.03 |
| Sensitivity | 0.71 ± 0.06 | 0.68 ± 0.03 | 0.78 ± 0.05 | 0.74 ± 0.03 |
| Specificity | 0.71 ± 0.05 | 0.66 ± 0.07 | 0.77 ± 0.04 | 0.67 ± 0.04 |
| Precision | 0.53 ± 0.04 | 0.52 ± 0.05 | 0.64 ± 0.05 | 0.52 ± 0.03 |
| Recall | 0.71 ± 0.06 | 0.68 ± 0.03 | 0.78 ± 0.05 | 0.74 ± 0.03 |
